# Supplementary material for: Programming molecular topologies from single-stranded nucleic acids
Source: Nat Commun. 2018 Nov 2;9:4579. doi: 10.1038/s41467-018-07039-7 (PMC6214983; doi:10.1038/s41467-018-07039-7)
Supplement: Supplementary file 1 — Supplementary Information [file 41467_2018_7039_MOESM1_ESM.pdf]

Supplementary Information

Programing Molecular Topologies from Single-stranded Nucleic Acids

Xiaodong Qi†[1,2], Fei Zhang†*[1,2], Zhaoming Su†[3,4,5], Shuoxing Jiang[1,2], Dongran Han[6,7], Baoquan Ding[8,9], Yan Liu[1,2], Wah Chiu[3,4,5], Peng Yin[6,7], Hao Yan*[1,2]

*Correspondence to: fei.zhang@asu.edu, hao.yan@asu.edu

†These authors contributed equally to this work

This PDF file includes:

Supplementary Figures 1 to 19
Supplementary Tables 1 to 8
DNA Sequences

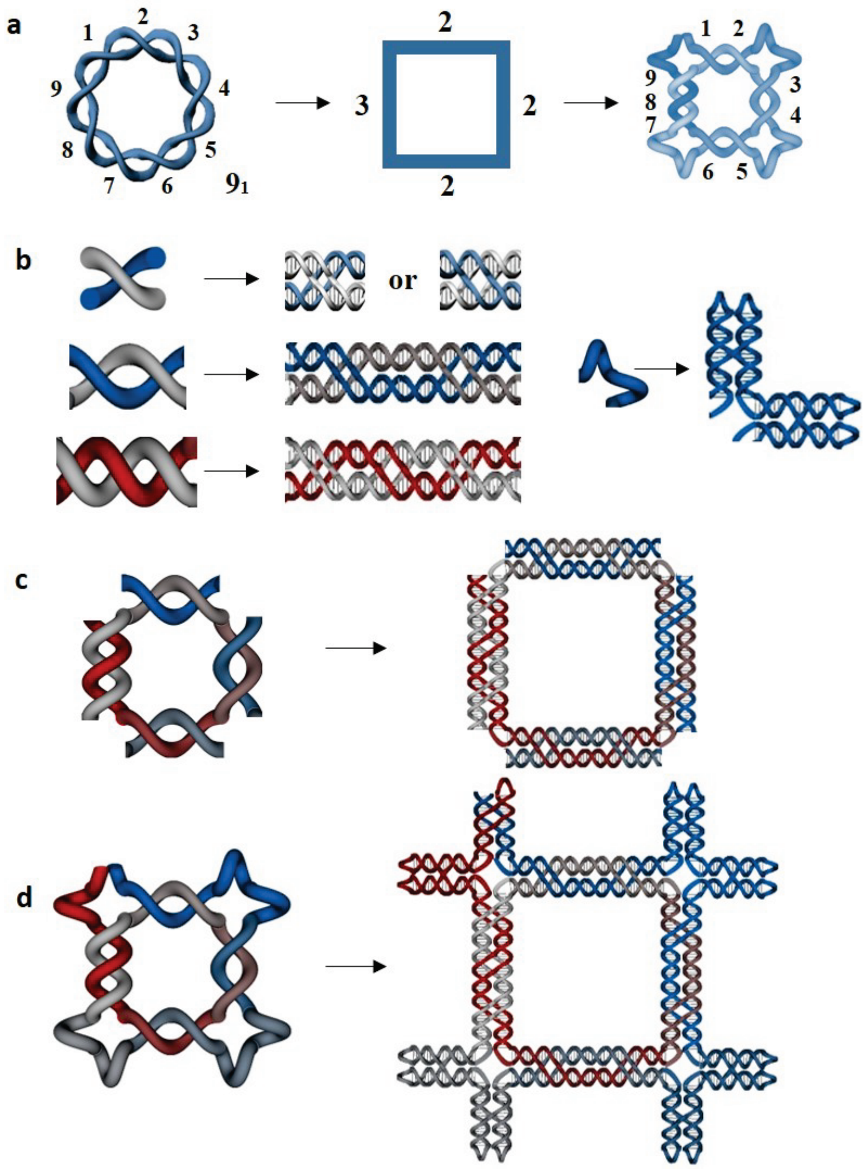

Supplementary Figure 1. The design of a DNA knot 9_1. a. The schematics show how to assign 9 crosses on a square geometry. As each edge has the same length, well-distributed crosses are preferred in order to maintain the stability of the DNA structure. **b**. Paranemic cohesion (PX) interaction, each contains two parallel crossovers with 4 bp or 6 bp. These base pairs are used to represent the cross in our knot design schematics. The distance between the adjacent PX crosses are designed as integer multiples of one DNA helical turn (10, 11, 21 or 32 bp). We also designed a small linking structure in order to connect DNA strands in each outer corner. **c**. An arrangement of each of the edges of the square with the corresponding DNA structures. **d**. Adding small linking structures at the vertexes finishes the design of the structure. The DNA strands in the inner corners are connected directly using poly T loops (T_4 is used for a 90 degree turn in the square). The outer corners are linked with the linking structure containing one PX.

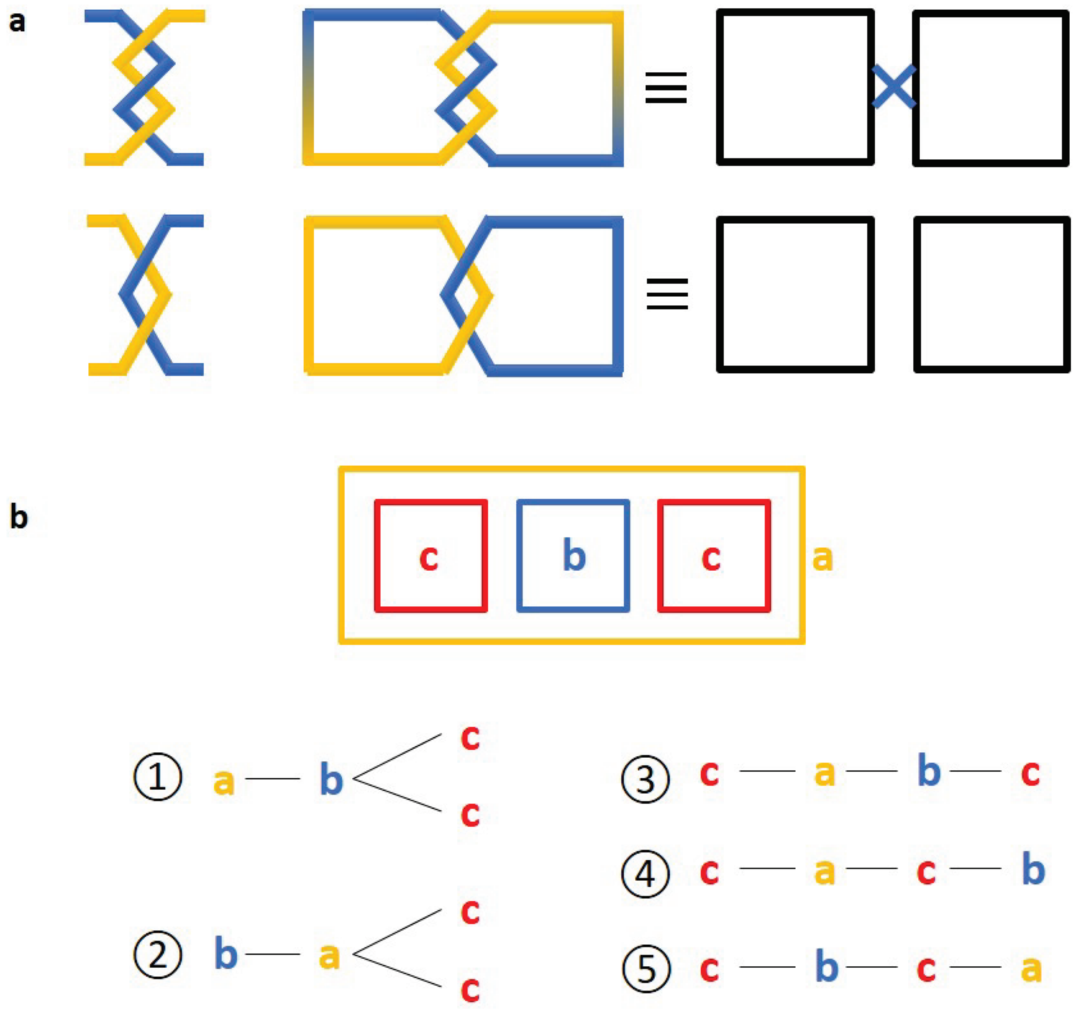

Supplementary Figure 2. A schematic of the folding pathway design. a. An edge with an odd numbers of crosses (1, 3, 5…) will allow the two loops that form the edge to connect into one large loop, i.e. to form a knot structure, while an edge with even numbers of crosses (2, 4, 6…) will produce a link between two separate loops. The schematics show an edge with 3 crosses that formed a knot of 3_1, and an edge of 2 crosses that resulted in a link structure called a Hopf link. **b**. In order to form a knot structure instead of a link, we needed to create an odd number of crossings among the individual loops and choose different orders of connections to link the four loops (*a*, *b*, *c*, *c*) to get the target knot. We named the loops involved according to their geometric relationships, and the two *c* loops are the same due to their symmetry. The largest loop *a* can be connected with either loops *b* or *c*, while *c* can only connect with loops *a* or *b*, not with the other loop *c*. We listed all of the possible orders of connections among the loops, which represent different folding pathways that could be used in order to form the target knot structures. Pathways 1 and 3 are branched and pathways 2, 4, and 5-8 are linear.

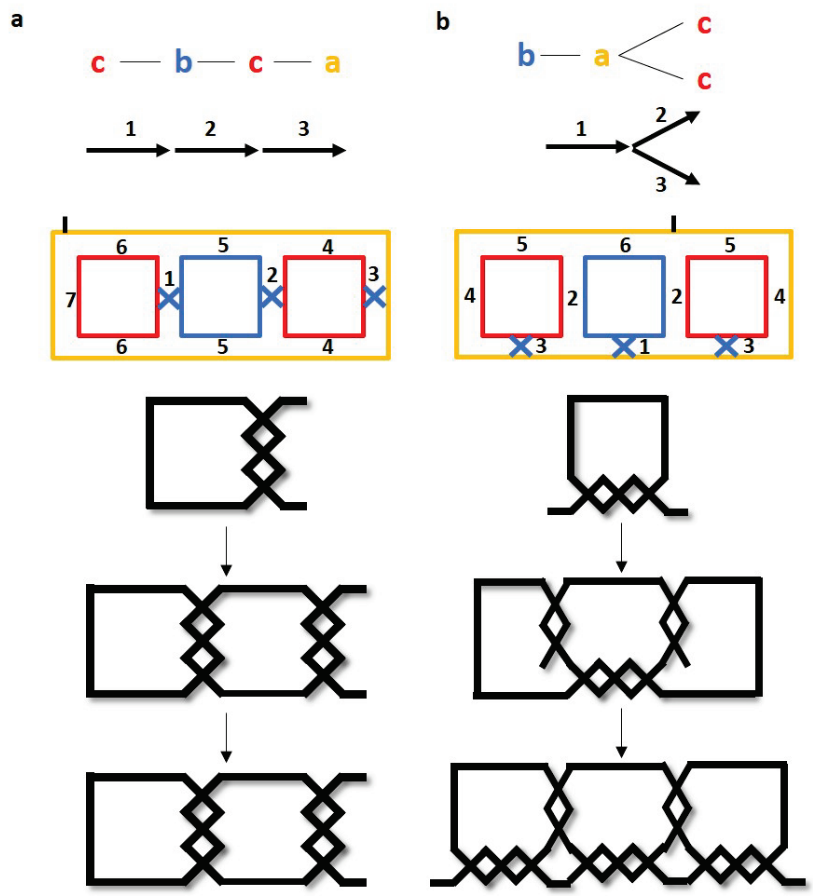

Supplementary Figure 3. A comparison between a linear folding pathway (a) and a branched folding pathway (b). According to the connection relationship between the loops, *a* to *c*, we assigned the edges with either 3 crosses or 2 crosses, (with the total number of the crosses being an odd number), to form the knot. The edges with 3 crosses are marked with an X. These edges should form earlier than the edges with 2 crosses, (due to the annealing step used and the difference in the strength of the paranemic cohesions involved). The pathways represent the order of the formation of the three cross edges, i.e. the formation of the corresponding loops. The direction of the linear pathway *c-b-c-a* (**a**) can be reversed as *a-c-b-c* without changing the relationships of loop connection. However, these two linear pathways are not equivalent. We selected *c-b-c-a* as a preferred direction because the two ends will not need to thread into any preformed loops during the early steps, which is when the unfolded strand is still long. For the branched pathway (**b**), the two ends need to be separated after forming the first 3-cross edges. Each end then travels individually and threads through a preformed loop (the central one) in order to create the 2-cross edges to form the loops on the sides. Therefore, the branched path is expected to be less favorable than the linear one because the formation of the 2-cross edges is expected to occur later than the 3-cross edges due to thermodynamic reasons. Among the linear paths, the best path should avoid threading through pre-formed structures when the unfolded strand is long. Due to these reasons, path 5 (illustrated here) is better than path 3 and 4 as shown in Supplementary Figure 2.

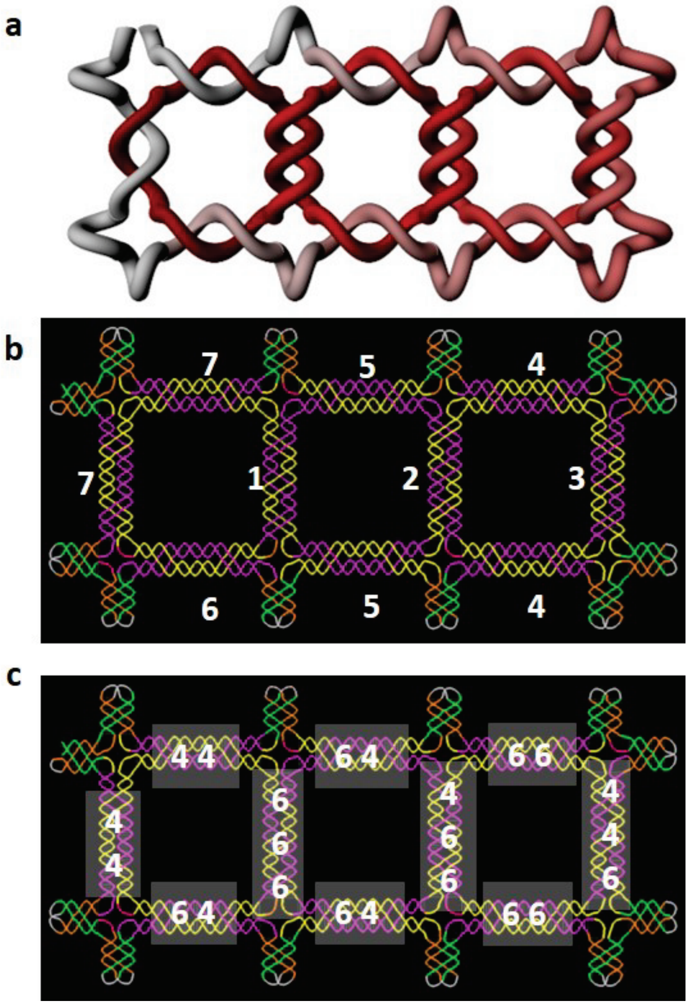

Supplementary Figure 4. The sequence design for the hierarchical folding based on the selected best pathway. a. A schematic of the cross section of the *c*-*b*-*c*-*a* folding path (as shown in Supplementary Figures 2-3). The ends of the partially folded dsDNA are located at the upper left corner. The gradual color change from red to grey represents the order of the looping. **b**. The folding order of all of the edges are labeled as steps 1 to 7. The edges that are marked as 1-3 are the 3-cross edges while 4-7 are the 2-cross edges. **c**. A DNA structure that represents the target topological geometry. The white numbers are the length (bp) of each paranemic cohesion interaction.

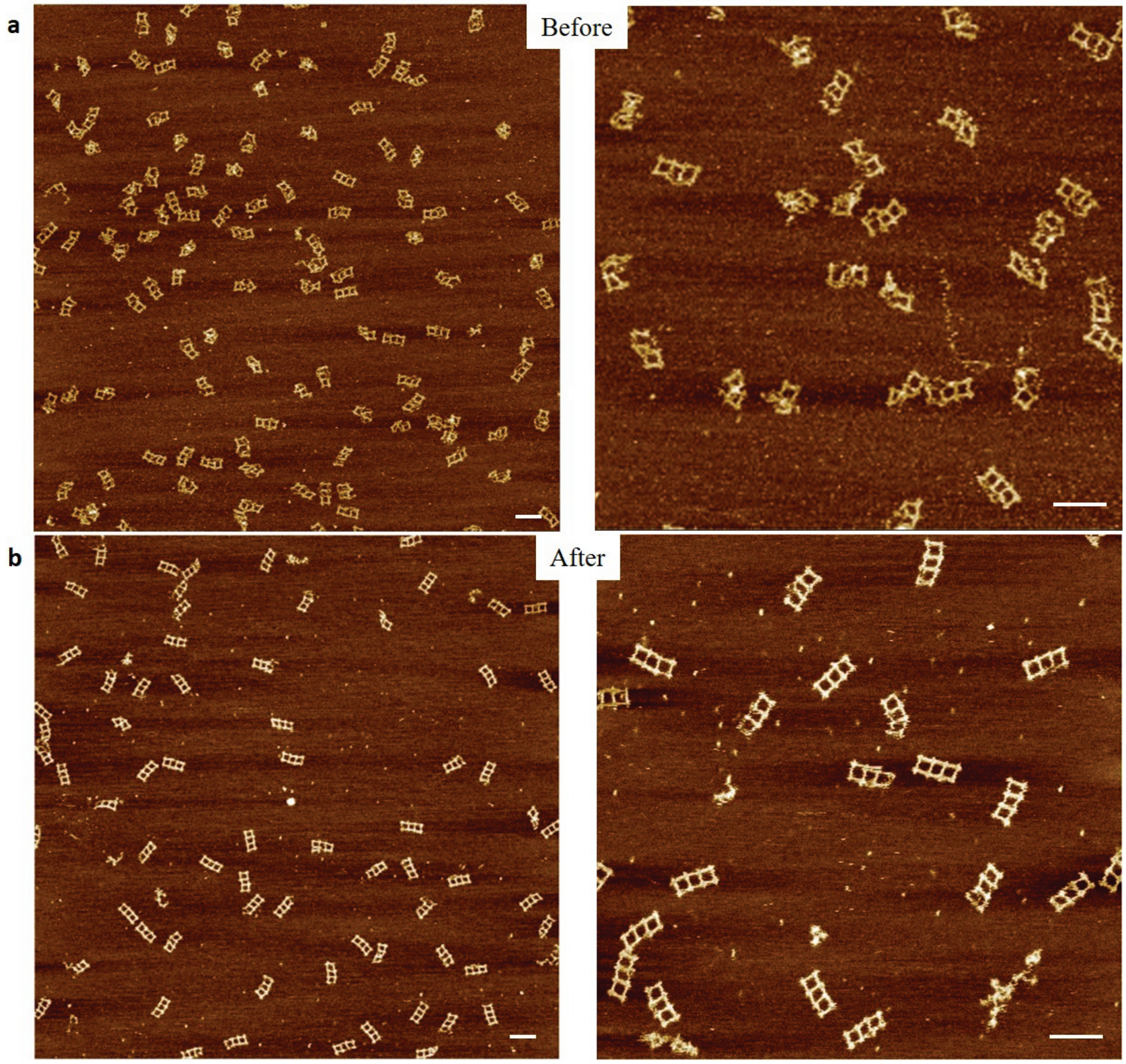

Supplementary Figure 5. A comparison between the yield of the three-square knot structure 9_1 and the different folding pathways, via the use of AFM imaging. With an unfavorable folding pathway (**a**), the folding yield is only 0.9% (2/221). With the best folding pathway (**b**), the folding yield is increased to 57.9% (124/214). Scale bars are 100 nm.

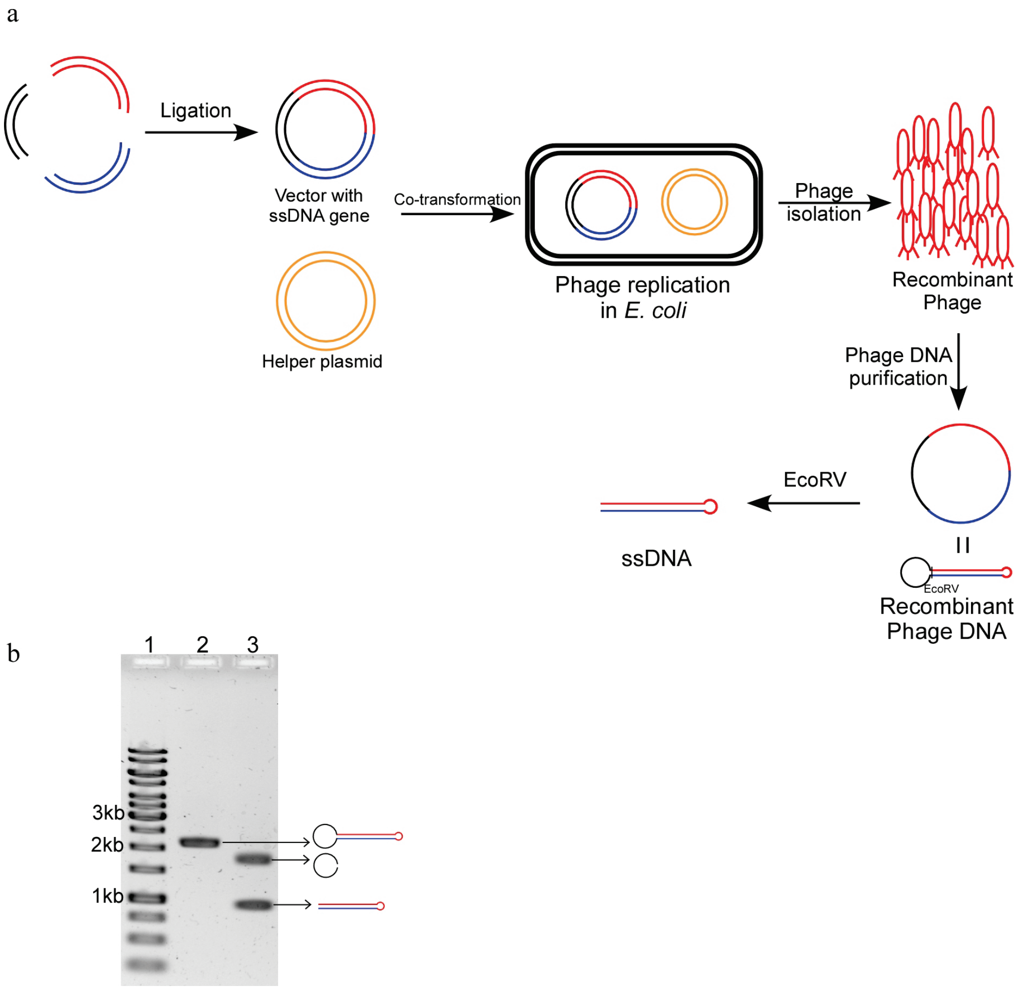

Supplementary Figure 6. The replication and production of an ssDNA by using a recombinant M13 phage. a. The ssDNA replication process. First, the two halves of the ssDNA gene (red and blue), were obtained by restriction enzyme digestion from a commercially synthesized plasmid. Then, the custom synthesized ssDNA gene was ligated into a phagemid vector pGEM-7zf(-) (black) by T4 DNA ligase (New England Biolabs) and co-transformed into *E. coli* DH5α competent cells (New England Biolabs) with the helper plasmid pSB4423.(*1*) During the phage replication, the ssDNA sequence (red and blue) was packed into the phage capsid as its genome. Recombinant phages were then harvested from the *E. coli* medium and the recombinant phage genomic DNA was isolated and purified. The EcoRV restriction sites were initially designed at the ends of the ssDNA and the phage DNA digestion by EcoRV restriction enzyme produced the ssDNA molecule (partially paired and folded into a hairpin, with the 5’ and 3’ ends meeting each other and the unpaired bubbles as paranemic cohesion sites). **b.** An example of the 1800 nt ssDNA purification by gel electrophoresis. Lane 1 represents the 1 kb dsDNA ladder. Lane 2 contains the purified phage DNA without EcoRV cleavage. After EcoRV digestion, the 1800 nt ssDNA molecule (lower band) is separated from the vector DNA (upper band) in lane 3. The 1800 nt ssDNA molecule runs slightly faster than the 1 kb dsDNA (2000 nt).

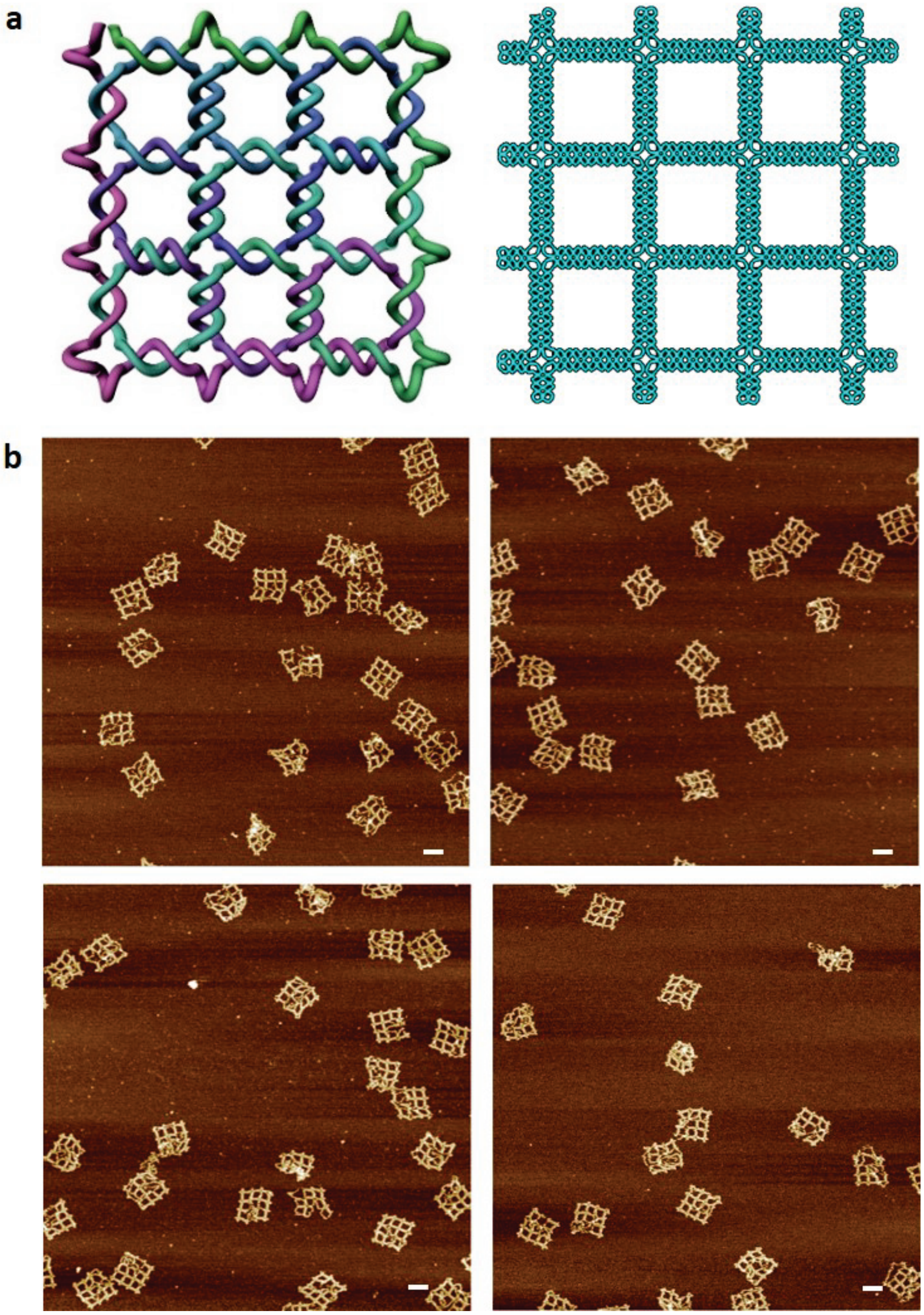

Supplementary Figure 7. The design and characterization of the 9-square knotted DNA structure. a. Design schematic. **b**. AFM images. Scale bars are 100 nm.

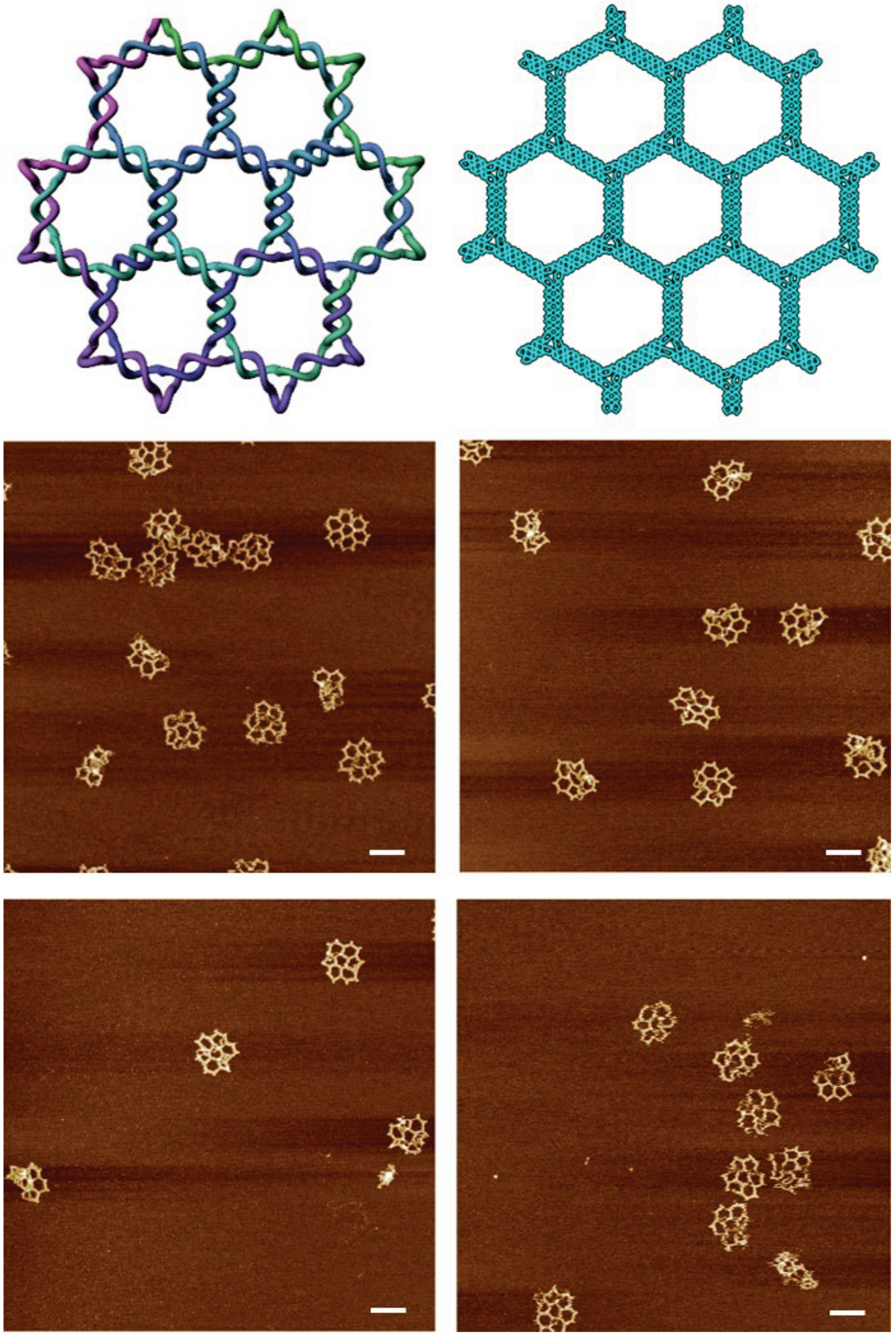

Supplementary Figure 8. The design and characterization of the hexagonally knotted DNA structure. Design schematic (top) and AFM images (bottom). Scale bars are 100 nm.

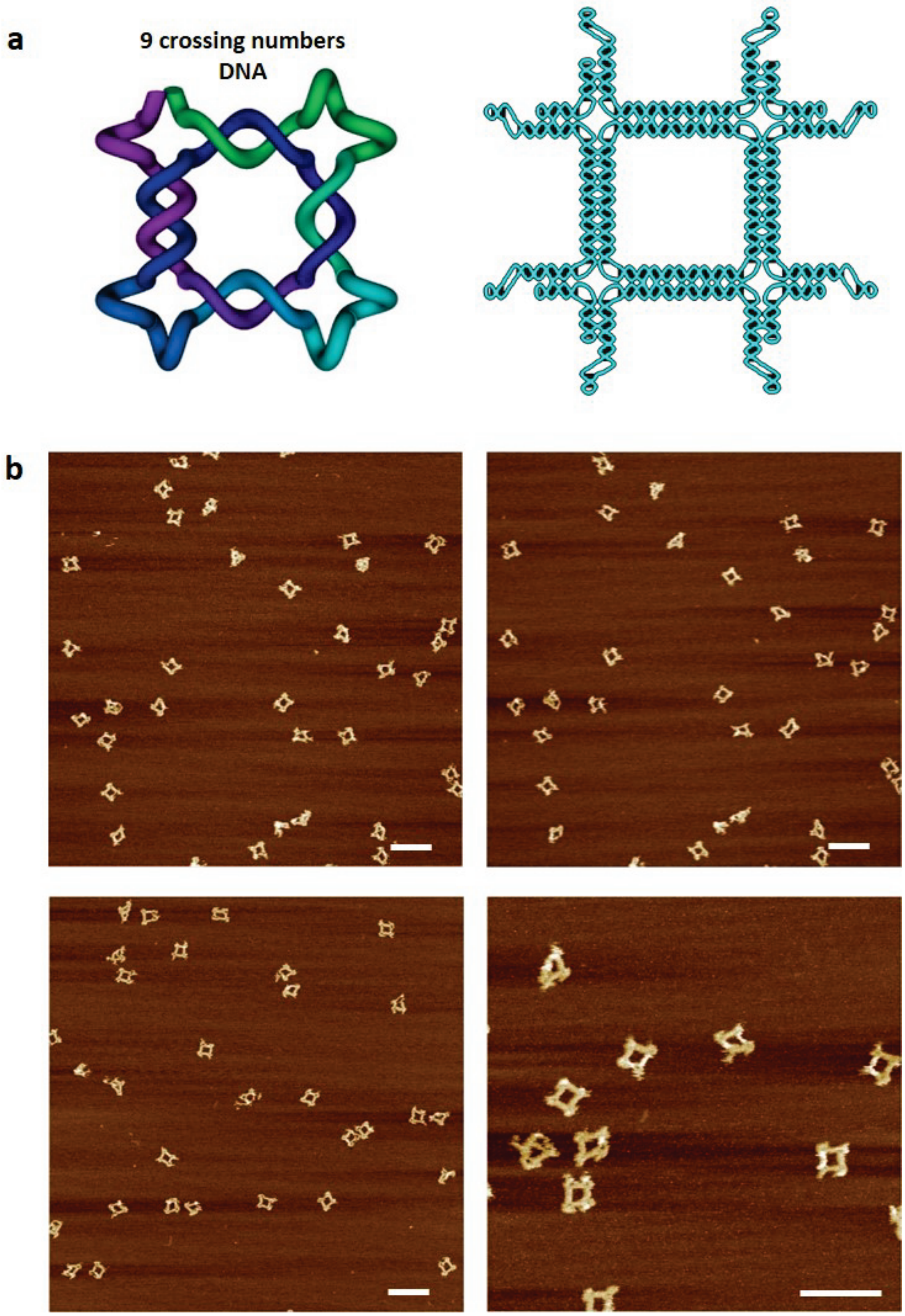

Supplementary Figure 9. The design and characterization of the square knotted DNA structure 9_1. a. Design schematic. **b**. AFM images. Scale bars are 100 nm.

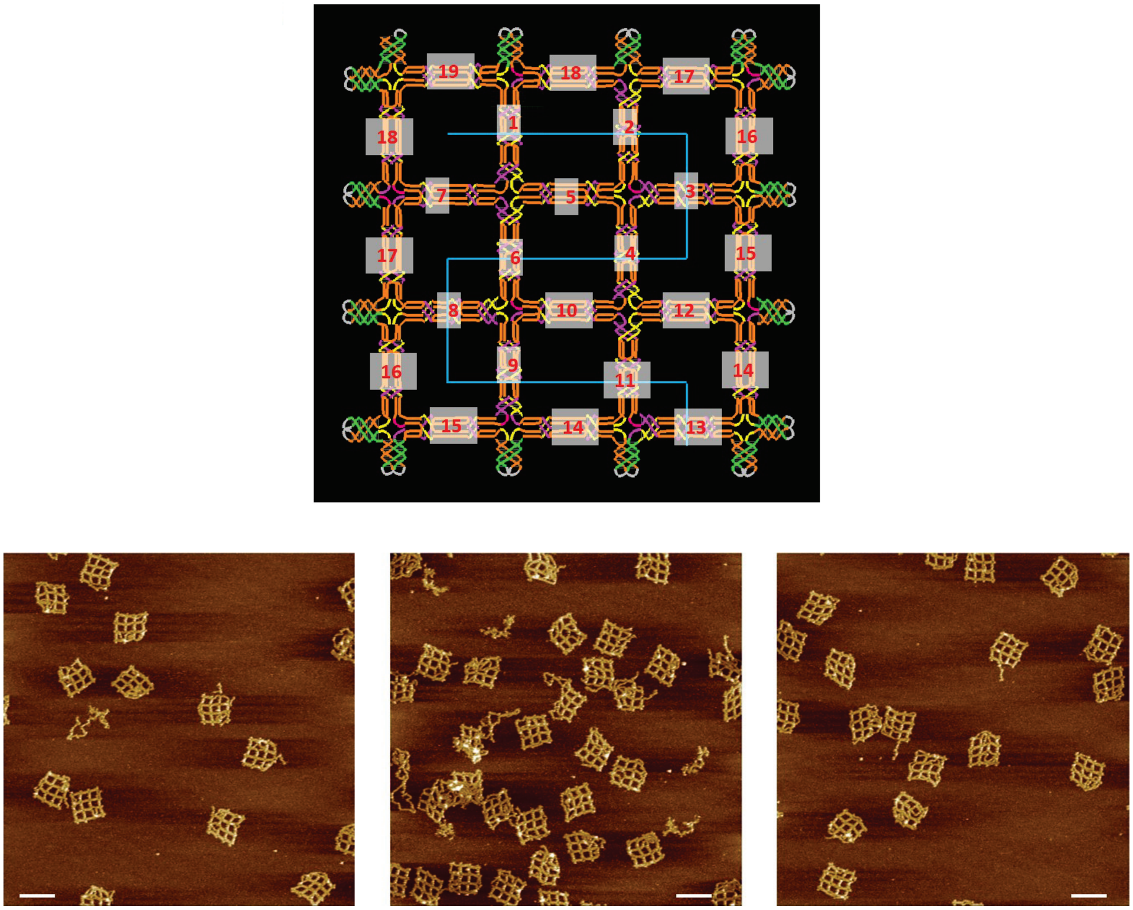

Supplementary Figure 10. The design and characterization of the 9-square knotted DNA structure with hierarchical folding. (Top) Folding pathway design. The numbers on the edges mark the anticipated order of the formation of the crosses on the edges, based on the designed sequences. **(Bottom)** AFM images of the folded knot structure (with 57 crossings). A majority (if not all) of the structures formed show some degree of errors. It seems that if the crossings in some of the earlier steps did not form properly, the crossings in the later steps could still form, but that the errors would be permanently trapped and there would be no chance of correcting the errors. Since each crossing may have a certain rate of error, with 57 crossings, the final product is expected to have a low yield. Nevertheless, the stepwise yield is quite high (>90%) with the overall structures having a high resemblance to the expected design. Most of the structures have more than half of their edges properly formed. The scale bars are 100 nm.

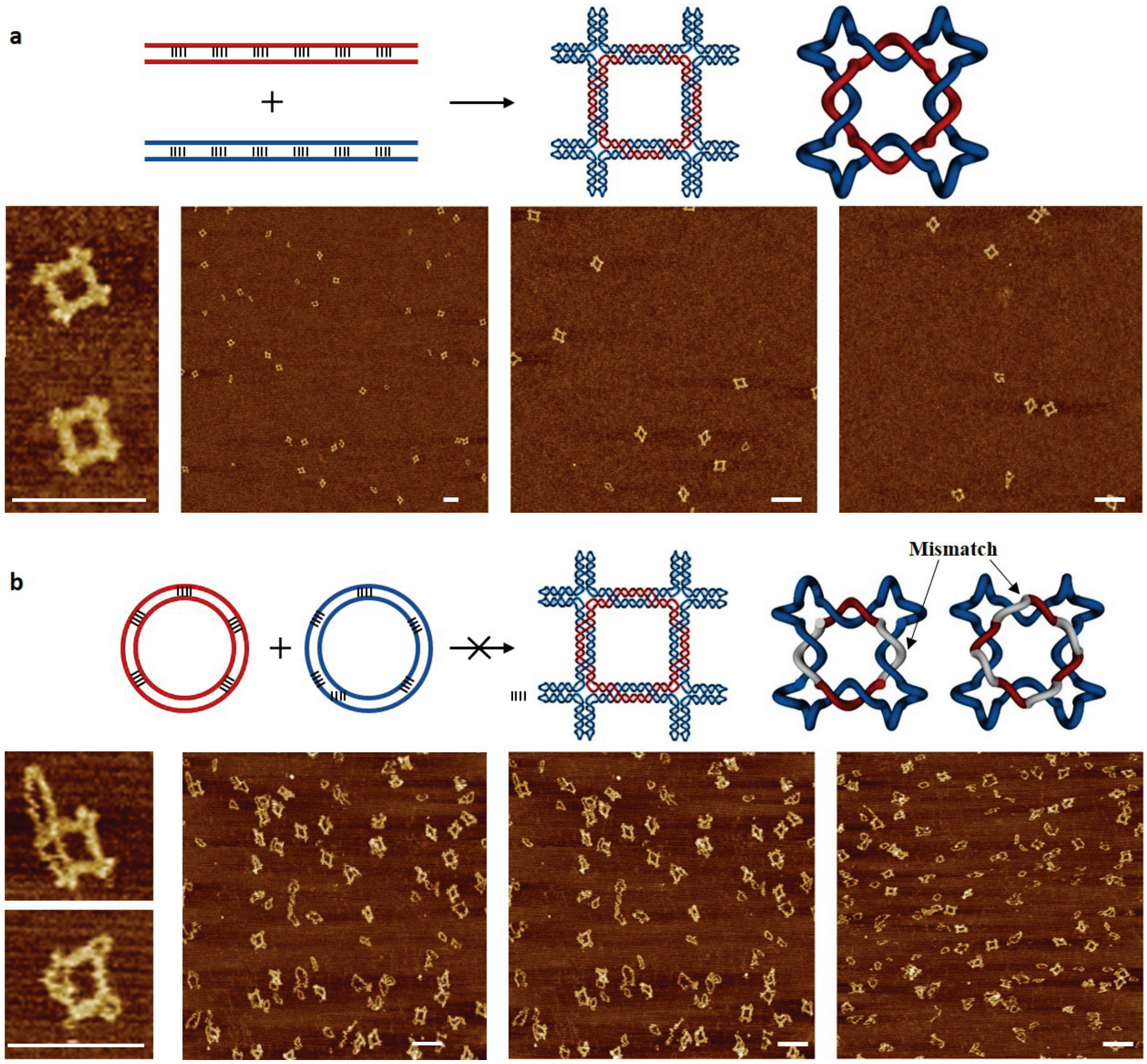

Supplementary Figure 11. Topological control experiments with linear and circular dsDNA. A DNA link structure with linking number of 8 was designed and constructed from four linear ssDNA. a. Two linearly annealed and partially paired dsDNAs (with internal loops for PX cohesions between the two DNA), can self-assemble into the designed structure with a high yield, as shown in the AFM images. **b**. After circularization, although the two dsDNA rings could still bind with each other partially through some of the paranemic cohesion interactions, extensive defects were observed in all of the structures under high resolution AFM. The two circular dsDNA molecules cannot form the correctly interlocked nanostructure. All scale bars are 100 nm.

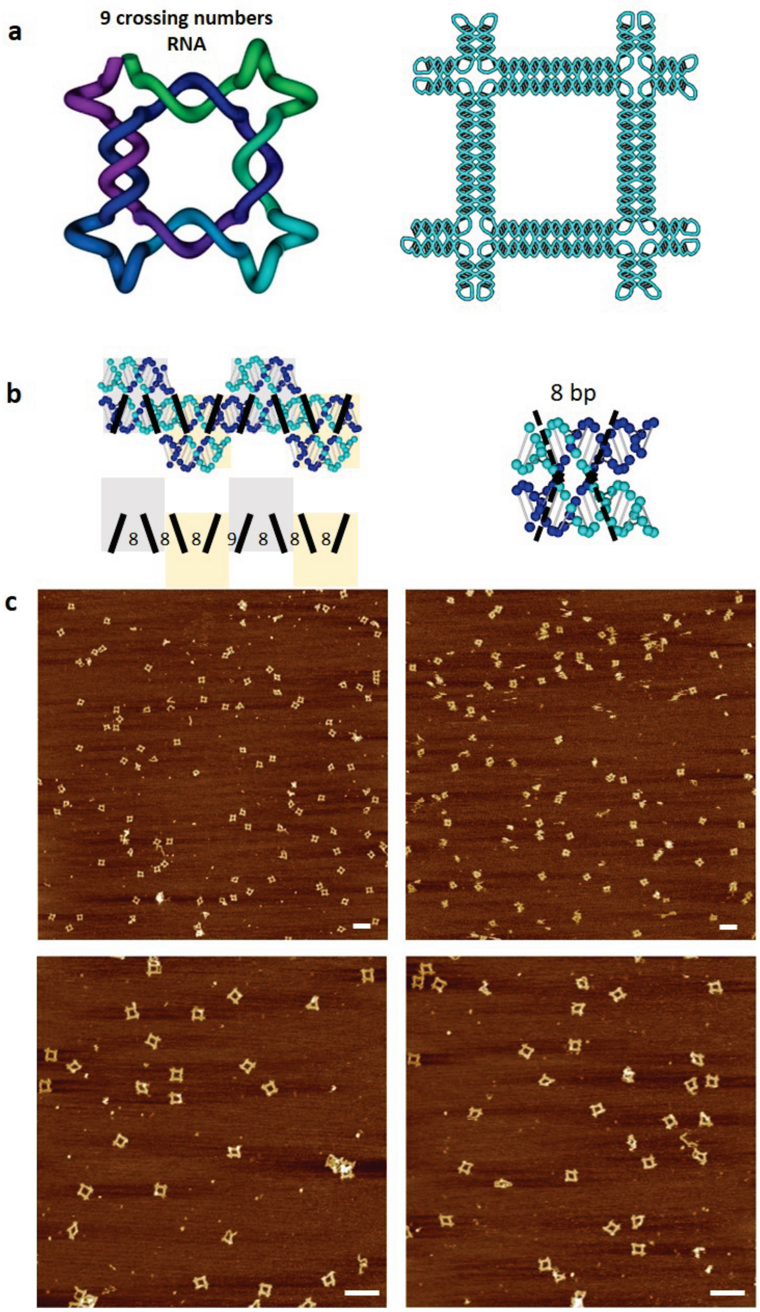

Supplementary Figure 12. The design and characterization of the square knotted RNA structure 9_1. a. The design schematics. An a-form double helix is used as the structural model. **b**. This image shows an RNA paranemic cohesion design that was based on A-form double helices. 8 bp was chosen as the length of the PX cohension for RNA; 33 bp was chosen as the length of the repeating unit (3 full turns). **c**. AFM images illustrating a high yield (~ 60%) of the expected structure. All scale bars are 100 nm.

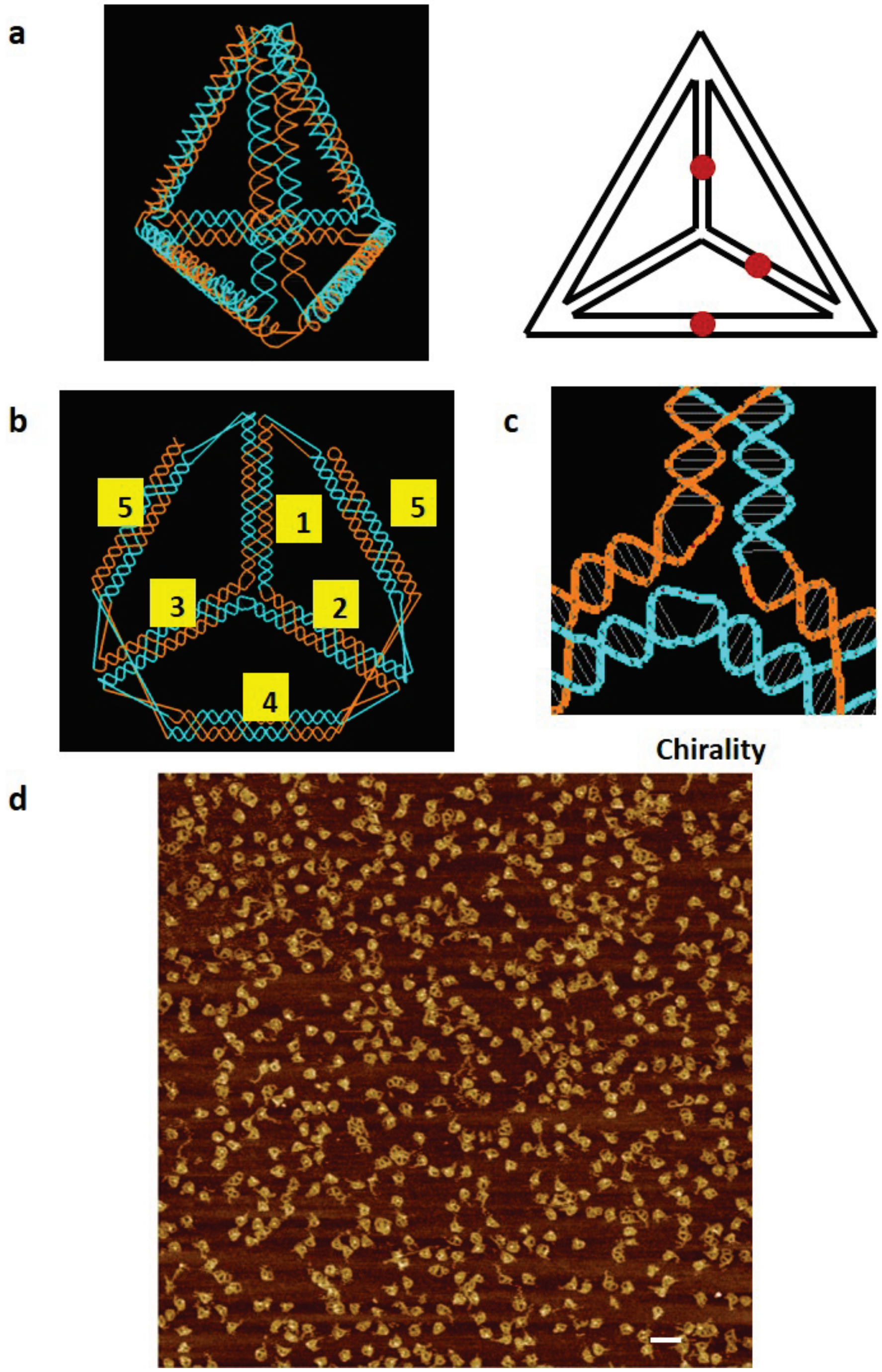

Supplementary Figure 13. The design and characterization of the tetrahedron knotted DNA structure. a. The design schematics of the folding pathway. In the middle 2D diagram, the red dots mark the edges of the tetrahedron that have the 3 crossing numbers. b. The number on the edges mark the anticipated order of formation of the edges. **c**. The vertexes all show the illustrated chirality. **d**. AFM image. Most of the structures in the image display the expected structure. However, some of the structures shown are missing one or two edges. The scale bar is 100 nm.

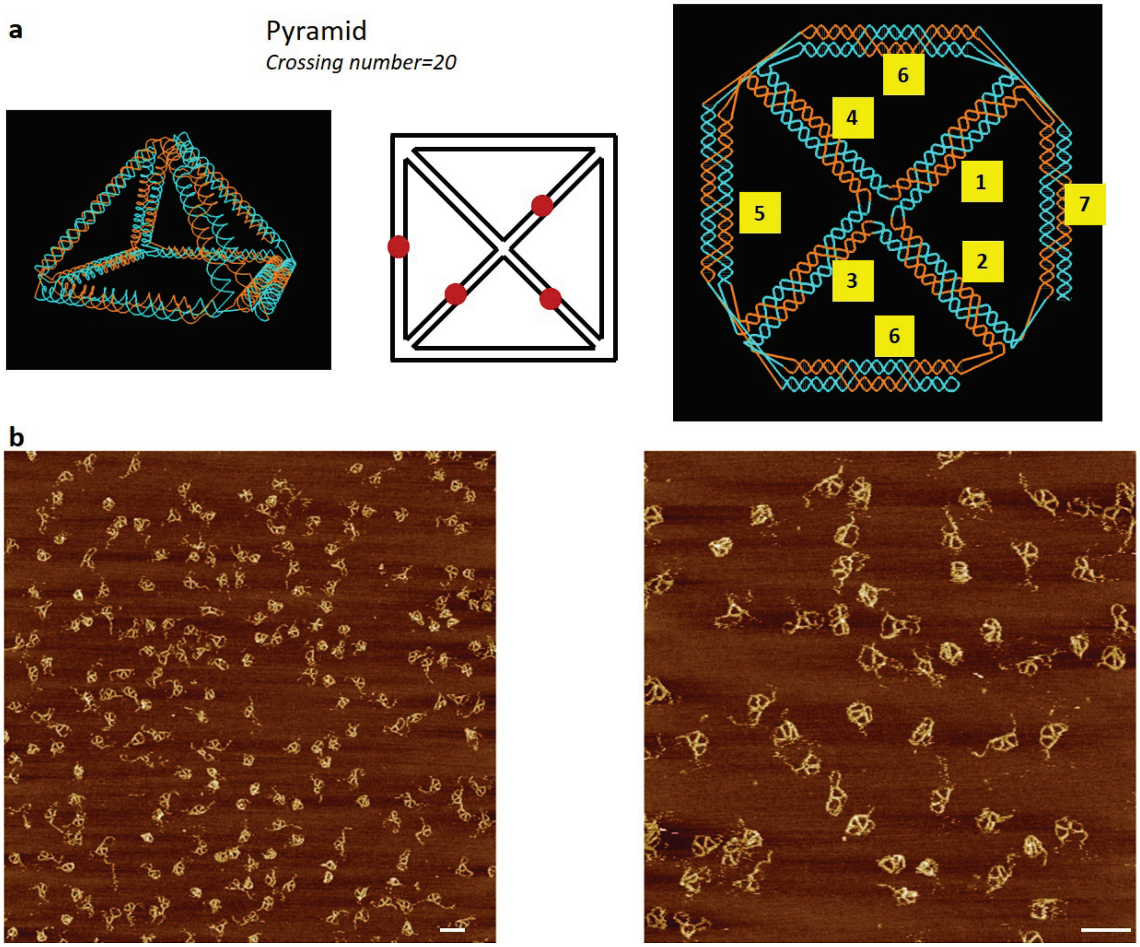

Supplementary Figure 14. The design and characterization of a pyramid knotted DNA structure (crossing number = 20). a. The design schematics of the folding pathway. **b**. AFM images. However, although many of the structures that are shown are distorted or broken, due to interactions with the substrate surface, a majority of the edges shown are well formed. The scale bars are 100 nm.

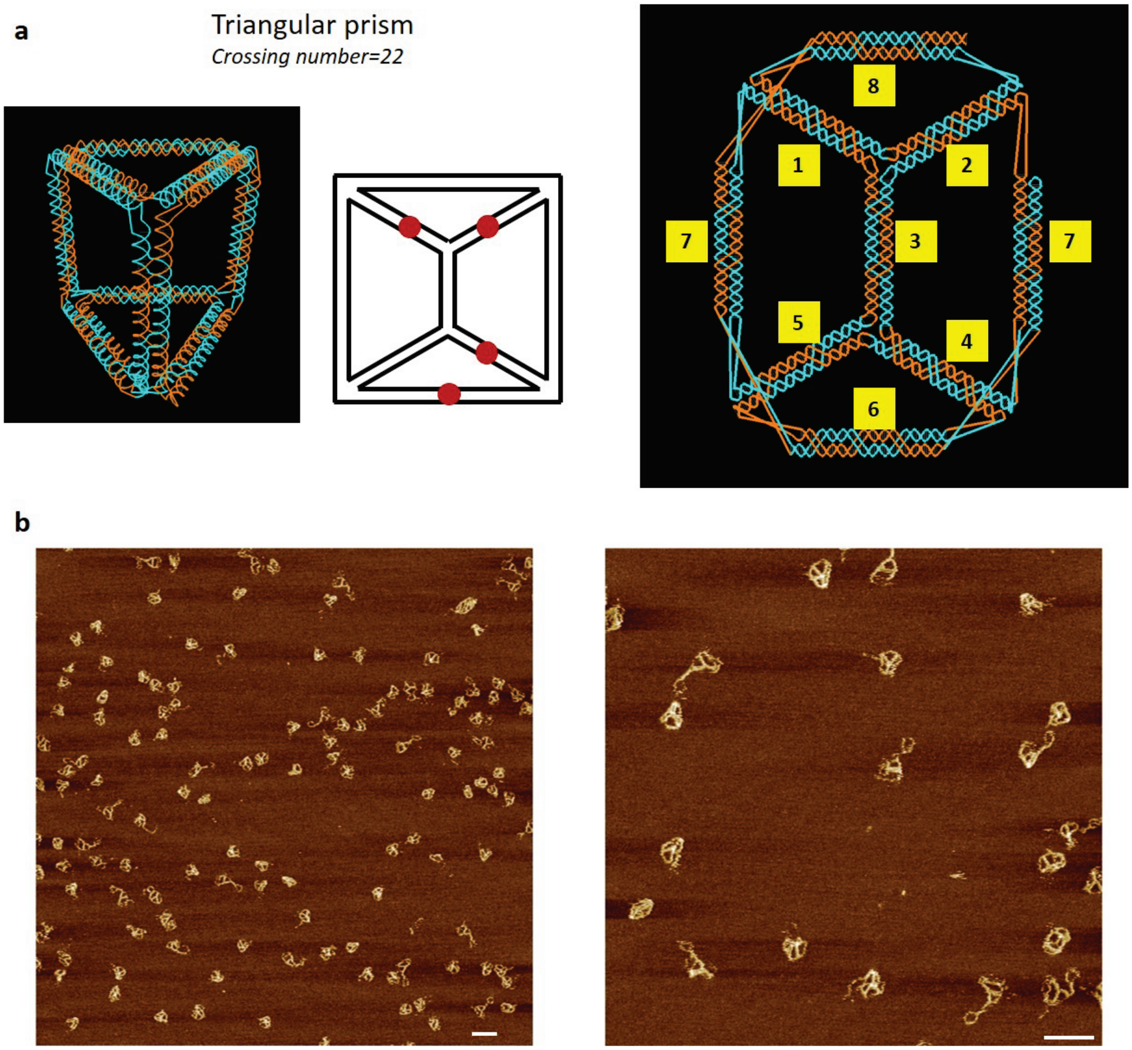

Supplementary Figure 15. The design and characterization of the triangular prism with the knotted DNA structure (crossing number = 22). a. The design schematic that includes the folding pathway. **b**. AFM images. The scale bars are 100 nm.

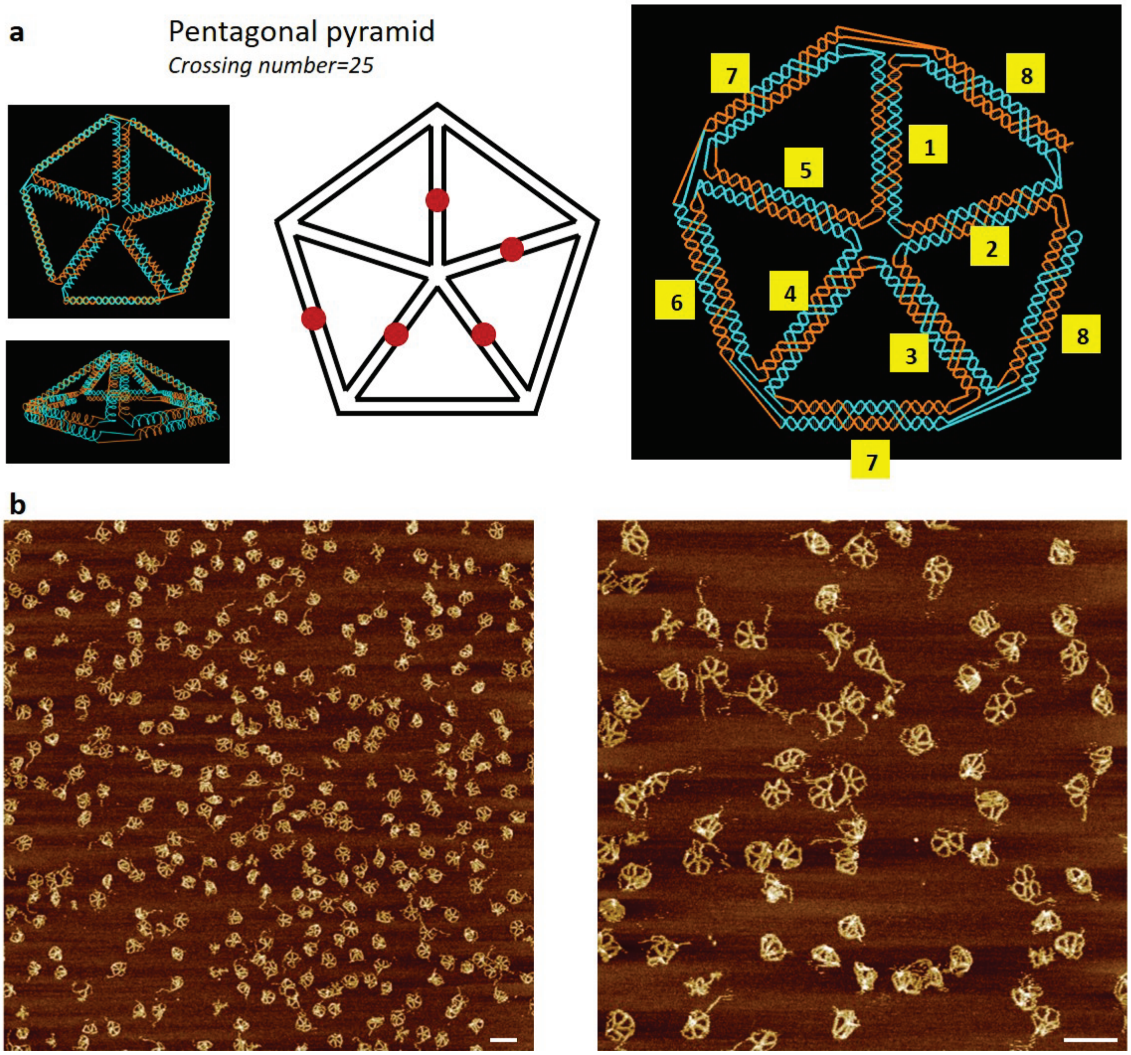

Supplementary Figure 16. Design and characterization of a pentagonal pyramid that has a knotted DNA structure (crossing number = 25). a. The design schematics of the folding pathway. **b**. AFM images. The scale bars are 100 nm.

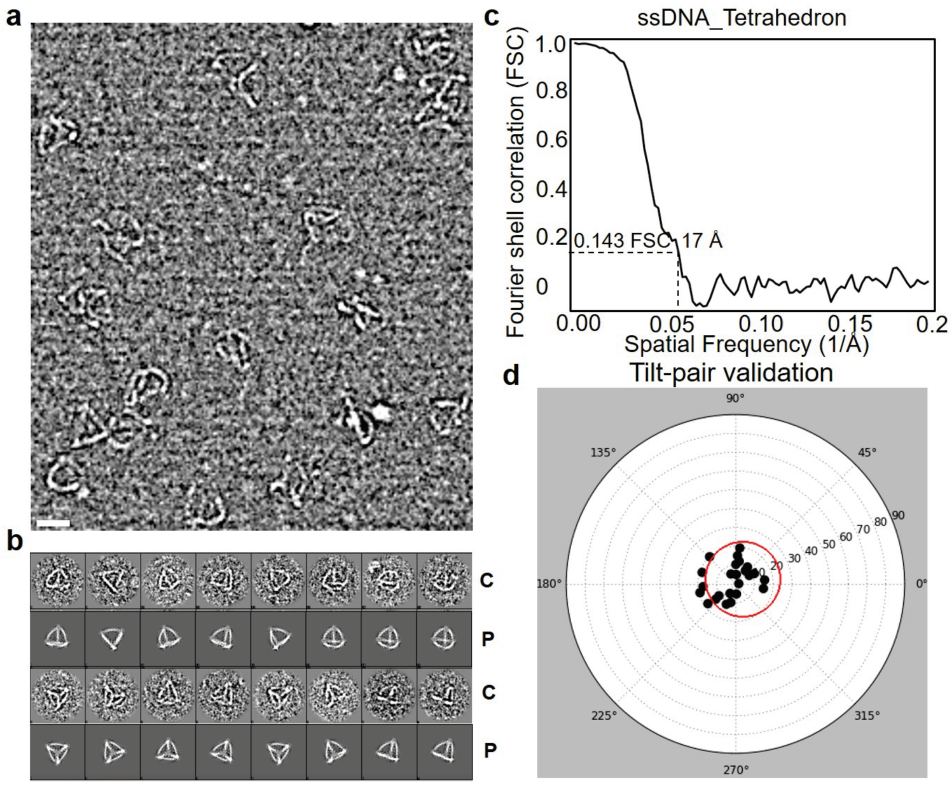

Supplementary Figure 17. Cryo-EM characterization of a single stranded DNA tetrahedron. a. This image shows a raw cryo-EM micrograph with visible particles. Scale bar is 20 nm. **b**. These images show the reference-free 2D class averages and projections of the final 3D reconstruction. **c** This graph is a gold-standard FSC plot for the final 3D reconstruction of the particles. **d**. A tilt-pair validation result. The red circle shows particle pairs that cluster around the experimental tilt geometry.

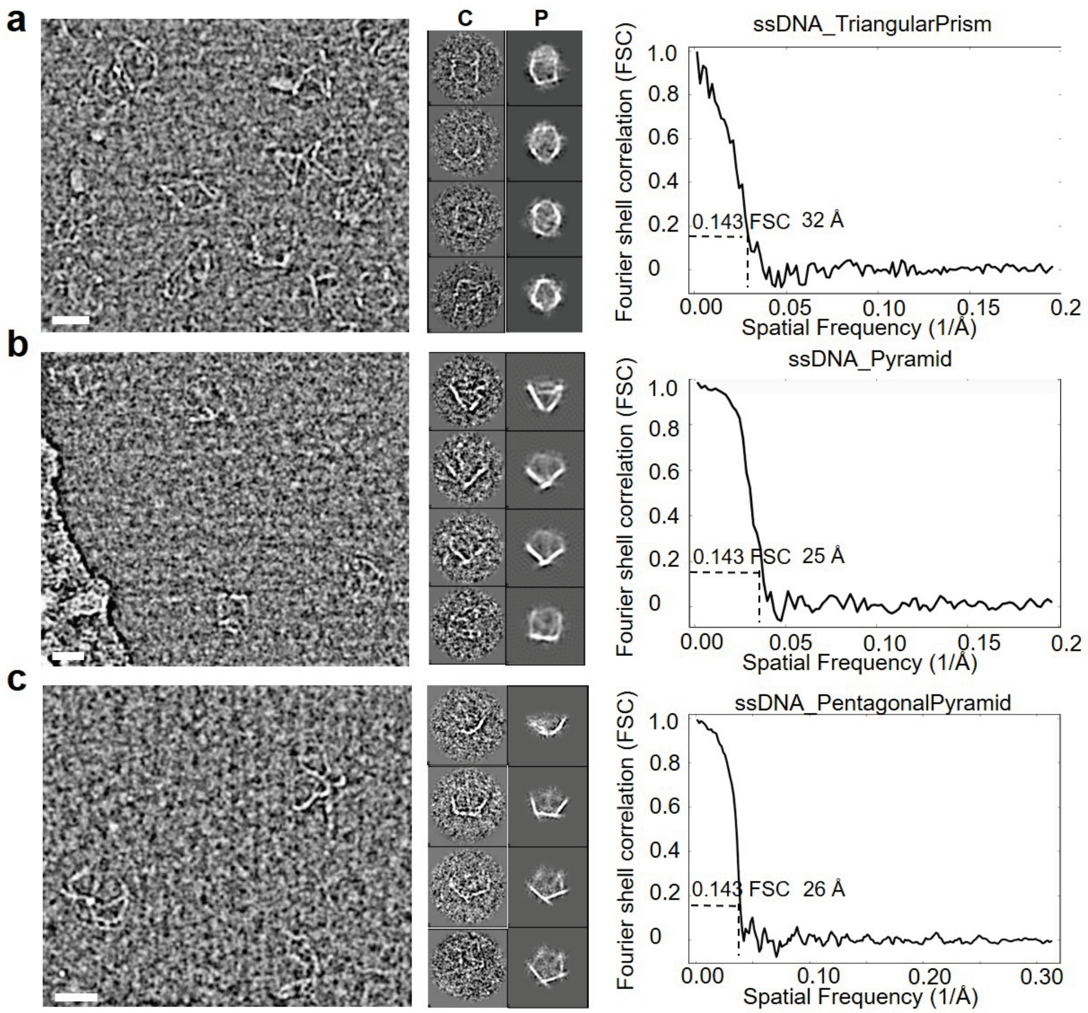

Supplementary Figure 18. Cryo-EM characterization of an ssDNA triangular prism (a), pyramid (b) and pentagonal pyramid (c). For each structure, a raw cryo-EM micrograph is shown with visible particles. There are also images on the right in smaller boxes, showing the raw particles. Furthermore, a Gold-standard FSC plot is also shown for the 3D reconstruction of these particles. All scale bars are 20 nm.

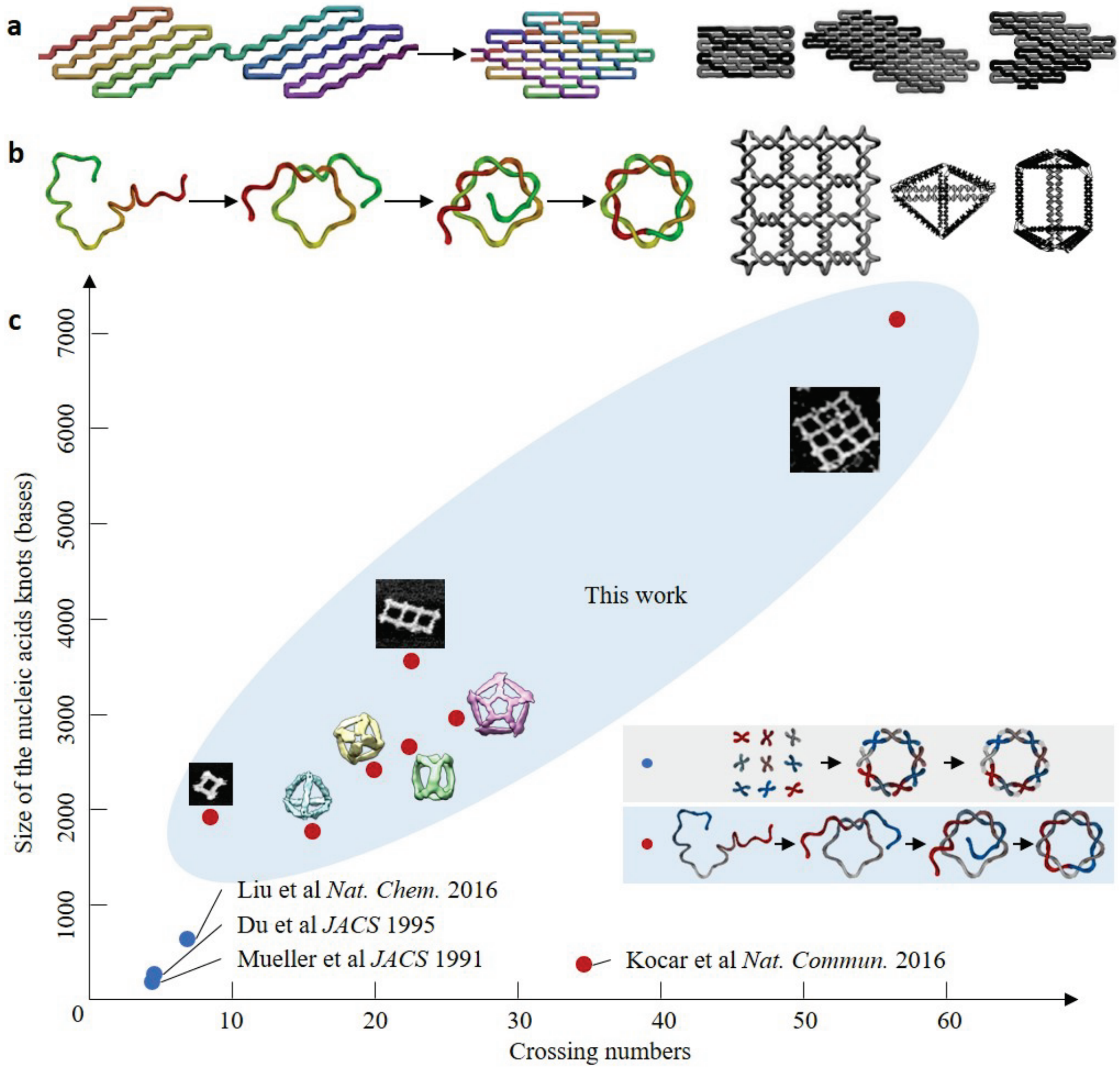

Supplementary Figure 19. Comparation of self-assembled topological DNA/RNA nanostructures. A knot can be constructed via two strategies by using DNA. The top two rows show that knot can be assembled by either connecting 9 right-handed X-shaped junction tiles together (blue dot), or by threading a single chain through itself 9 times (red dot). Prior to this study, it is a grand challenge to fold a single DNA/RNA strand into a complex topology with high crossing numbers and long length of nucleic acids in a programmable and controllable way.

Supplementary Table 1. A summary table of the design parameters for the structure in Supplementary Figure 4, including the number of bases in each PX cohesion, and the GC content of the PX cohesions in each edge.

Step	1	2	3	4	5	6	7
Cross number	3	3	3	2	2	2	2
length	666	664	644	66	64	64	44
bp	36	32	28	24	20	20	16
GC %	69	66	61	58	60	55	50

Supplementary Table 2. A summary table of the length of the PX cohesion, the number of the base pairs involved, and the GC content of the base pairs for the structure in Supplementary Figure 10.

Step	Length	bp	GC%
1	666	36	67
2	466	32	66
3	466	32	62
4	446	28	64
5	66	24	67
6	444	24	63
7	66	24	58
8	444	24	58
9	444	24	54
10	66	24	54
11	444	24	54
12	66	24	50
13	444	24	50
14	46	20	60
15	46	20	55
16	46	20	50
17	46	20	45
18	44	16	50
19	44	16	44

Supplementary Table 3. A summary table of the design parameters for the structure in Figure 3.

Step	1	2	3	4	5	6	7
Cross#	3	3	3	2	2	2	2
length	666	664	644	66	64	64	44
bp	36	32	28	24	20	20	16
GC%	69	66	61	58	60	55	50

Supplementary Table 4. A summary table of the sequence design parameters that facilitate the folding order of structure in Supplementary Figure 13.

Step	1	2	3	4	5
Cross number	3	3	2	3	2
length	666	664	66	444	46
bp	36	32	24	24	20

Supplementary Table 5. A summary table of the sequence design parameters that facilitate the folding order of structure in Supplementary Figure 14.

Step	1	2	3	4	5	6	7
Cross number	3	3	3	2	3	2	2
length	666	664	644	66	444	46	44
Bp	36	32	28	24	24	20	16

Supplementary Table 6. A summary table of the sequence design parameters that facilitate the folding order of structure in Supplementary Figure 15.

Step	1	2	3	4	5	6	7	8
Cross number	3	3	2	3	2	3	2	2
length	666	664	66	444	66	444	46	44
bp	36	32	24	24	24	24	20	16

Supplementary Table 7. A summary table of the sequence design parameters that facilitate the folding order of structure in Supplementary Figure 16.

Step	1	2	3	4	5	6	7	8
Cross number	3	3	3	3	2	3	2	2
length	666	664	644	644	66	444	46	44
bp	36	32	28	28	24	24	20	16

Supplementary Table 8. Tilt-pair validation of single stranded DNA tetrahedron

# Total particle pairs	49
# Particle pairs in cluster	15
Fraction in cluster (%)	30.6
Mean tilt angle (^{o})	9.47
RMSD tilt angle (^{o})	4.00
Mean tilt axis (^{o})	42.4
RMSD tilt axis (^{o})	54.9
Experimental tilt angel (^{o})	9.9

Sequences:

9 crossing number square knotted DNA
ATCCAGGAAGGGCTATGGTTTTCATCGAAGATAGACAAATAGACAGCATGCC
AATGATGATCAGAAGAGGACGAGTTTTGGCCATATCTGGCATGTTTTTCATGC
CGATTCTATCTGAGTTCGCCAACCTACTTTTTGTAGGTCGAGGAGAGCTTTTA
GCTCATCGAACTCTTACCAGTCATCTTATTTCCCAGCAATAACGAGGTTGGGT
TTTAGGACTTGCTTCGACTAGGAACGGGAGGGAGAAGGGAACGAGATACTCG
TAGATTTTGTTGACCGAAACAAACCAAACCGCAGCTACGACGCACCATGATG
GTATCTCGATTTTAGCTCAGGGCACTAGTGGTAGGTAGTGGTGGGGTGGCGA
ACTACCTGTCTATCTTTTCCTCAAGACTAGAATCCTCATCGTGATGAGTACAG
GAACAGTAGGACAGCTGATTTTGGATTCCCAGAGTGACTTTTTGTCACTAGGC
ACCTCAGCGAAATCTATCTCGGTTTTTCCGAGAACAGTACCAGTTTTAGGCTC
GCGGTTCTTGGAACCTTGGCAGTAGACAACCTTTCCAGGGAACGTGCTTTTGA
CCTAACTTGGATGCTTTTTGCATCCTGTTAGTAGCGGCCTCCGTGACGTAGTT
TTTCTACGTAGCTGATGGATTTTGTACCGCTGCAGTCTGCTACATCAGGGACG
GACTGATTCACCTCTAGCTCACATTTTCTAGCGGGTGGTGAGCTTTTTGCTCA
CGAGTGGAATTCAACGGCCCTTTCAATCTTTTTGATTGAAGCATCCGTTGTTT
TAATTCCACTCGCAGTACATCTATGTGCTCAGTCTCCGTTTTTCGGAGTAGTC
GCACATAGATGTACTGCCACCCGCTAGTGTGAGCTAGATCATGATCAGTCCG
TCCCTGATGTAGGTTTGTGCAGCGGTACTCCATCAGCTAGTAACACGCAAGTG
GTACCTCCTGGCTTTTTGCCAGGCTAAGCCACTTGCGTGTTACTCACGGAGGC
CTTTTGCTACTAACAGCTTCGTTTTTCGAAGCAAGTTAGGTCGCACGTTCCCT
CTCGTGGTTGTCTACTGCCAAGGTTCAGTCGACCGCGAGCCTCTGGTACTGTT
GAAACTTTTTGTTTCATAGATTTCGCTTTTTGAGGTGCCTACACGATCTCATCA
ACGCTAAGGCCACTTTTTGTGGCTACCTCGTTGATGAGATCGTGTCTGGGAAT
CCTCAGCTGTCCTTATTGTCCTGTACTCAGATGAATGAGGATTCTTCGATTGA
GGGATAGACAGGTCTGATGCCACCCCACCACTACCTACCTTGTCTGCCCTGAG
CTTCGAGATACCAGGTGAGTGCGTCGTAGCTGCGGTTTGCAGACTTTCGGTCA
ACTCTACGAGTATGGAAATCCCTTCTCCCTCCCGTTCCTCAAGAAAGCAAGTC
CTCCCAACCTCGTACTGTCTGGGAAATAATCACGCTGGTAAGAGTAGTCTGA
GCTGCTCTCCTCGAGTAGCTCGTCGACTCTAGTCGGTGTGTTTTTCACACCTC
AGTGAGTCGACGAGCTACTTGGCGAACTCTTTTAGATAGAATCGCTCTCTTTT
TGAGAGCAGATATGGCCCTCGTCCTCTTAGTTCCATCATTGGCATGCTGTCTA
TACTAGTATCTTCGATGCCATAGATGCTCCTGGAT

23 crossing number 3-square knotted DNA before hierarchical design
ATCACCCTTGGTCTCAGACGGATCAATCGCTGTGTTACTCGTACGGGCGATTA
CAGATTCACTGCGACACCTGGGAGATGCCGACTCCCATGACCAACTTTTTTCC
ACGTCATGGTCTTGTTTTCACCATACGTCCCCTGTTTTTTGTCGAGACGTTTAG
ACTCAACGCCCGGCACTCACGACGTGAGAATGGGCCTCTACGTCCTGCTCGT
CGTCTACACATGTGCTACACCGTGTTTAGTTTTTTGGAGGCACGGTGCAGCTT
TTAGTACATGTGCCCAGAGTCTTTTGCCGTCGTTGTAGCAGCAATCATGTTGT
CAAGCATGTAGCTTTTACGGAGAGATCCGTTCTTTTTTCGGTCGATCTGTTTCC
GTACCTGCGCTAGGCCGATGTGATGCCTGGCTCGTTACTTTGCACTGTCCGAC
AGGTTATTCCGAGTTCGGTTTACATGTTTTTTGCCTGAAACCGACTCGTTTTAG

TAACACGAATAGGCTTTTTTGCGCTTTCGTCAGGAGGCAGTCTATGAACTGGC
TTTGGGACCTACCATGCGGCTCCTGAAGCTGAACGACACGGACCTTCGGTCG
GTGTAGATTGTCTCGGTTGTCATCCATATGGCACGGAAAGACAGCGTGTTACC
CACCGATCGTTCGAGCAGCTCAACCGGATTGGAGGATCAGCTCGCTGGAGTT
CTGGCGACGCACGACGCACCTTTTAGGACAACACCGATTAGGCCTTAAAGCT
ACTTACGGTATCTGGGCATTGTGGTCTACTTCCAGGTGTAGGTCCCTAATCCC
GGCGTGCTACAAGCCCATGAACAGGGCCTTAGGCGTCCACCTTCCCTTTGGA
AGATTTCCAGCGCAGCGACACAGCGATCCCGTGGTTAGTAGGTGATCTGTTG
TCCGTGAGACGTACAAGGTATTACCACTCGACGAAAGTGACATATCCCCAGA
AATGTTCTGCATTTAGTATCCCTGGATGCTTGCTGTCTTCCATGTGCAATGTA
AAACCGCTGGAGTTGGCTTTGGTGTCGTCAGGTAAGCGTTTGGCAGAGGCAA
GAGAGCAAATTCGCCATAAGAGAGATCTCGCGAGGTATGAGGGTACCTTGCG
CTCTTAGCCATGGTGCACCTCACACTTCACTCTCTGGTGGTTCGCAGAGGCCT
GAGCTGTTCGCACGTCCGCTGCACCTCGTGACCACACTTGTTAGGAATCGAAT
GGGACGACTAATGAGCCTTTGAAACTGGTTGACGTCTCCAGAGGACGTGTCT
AAGGCTGAGGCAATGCTGGCTGACACGGACAACCTGGTCACTTGCGCTCCGT
CGTCACGACTCTTGGGGCCATGAGGTTGACGAGTTTTTGCCTCCAACCTGCA
GCTTTTCAAGTTAAGTGAGACCTTTTTCACAGCACTTCATCGCCAACTCGCC
AATGCGACAAGCTGTCTCTGGAAAACGCACATACGTTGGAGTGGATGGAGAT
TCCGTAGACGTATGACTGTTTTTCAGTGATACGTGATACTTTTATGGGTAGG
AGAAGTGGTTGAGTTCGTACCATTTGCCAGTCTCGTCTTCCTCCAGACCCTAC
GTTTTCATATGACGTCAGTACTTTTTTCGTGTGACGTGGTTTCCCCGACACTGG
AGTCGCTTTGGCAAGGGGTTGTGGCAAATCGTTAACGGTTTGCACGCAACAT
CTGCATCATGGCAACCTTTTTTCGGTTCCATGAAAGAGTTTTCTGAGTGACCA
GTCTAGTTTTTCTAGACGAACTACTCAGCTCTTGCTCTTAACCGTTTTTTGGTT
GAAGAGCTGCAGTTTTATGTTGCGTGCAAACCCGGGACGATTTGCCACAACC
CCTTGCCAAAGCCCAACCAGTGTCGGGTTTTGAAACCTGCCAACACGTTTTTT
GTACTTGGCACATATGCGTAGGGTCTGACTCAAGACGAGACTGGCAAATGGT
ACGAACTCAACGAGGAGTCCTACCCATGTATCCCCTCACACTGTTTTTTCAGT
CTGAGGGCTACGTTTTGAATCTCCATACGCTGCAACGTATGTGCGTTTTCCAG
AGACACGAGACCGCATTGGCGAGTTGTTTTGCGATGGCCCACTGTGTTTTTTG
GTCTTGGGCAACTTGGCTGCGAAAGCGAGGCTTTTTTCTCGTGCTTTCCATGG
TTTTCCCCAAGAGTTCGTTCGACGGAGCGCACCGCACCAGGTTGTCCGTGTCC
AGTAGCATTGCCTCTTTTAGCCTTAGACGTCGGATCTGGAGACGTCAACCAGT
TTCAAAGACATCGTAGTCGTCCCATTCGTTTTATTCCTAACAGCCAGAGTCAC
GAGGTGCAGCTCCTCCGCGAACAGCTCGCTGCTCTGCGAACCTTTTACCAGA
GAGTCTCCTCTGAGGTGCACCATGGCTAAGAGCGCAAGGTACGAGTATACCT
CGCGATTTTGATCTCTCTTCCCTGTAATTTGCTCTCTTGCACGCCGCAAACGCT
TACCTGAACCTGGCAAAGTTTTCCAACTCCAGACGACGTACATTGCACATGG
AAGACAGCAAGCATCCAGAGTGACTAAATGCAGTTTTAACATTTCTGGGGAT
ACGCAACTTTCGTCGAGTGGTAATACCTTGTACCACACACGGACAACATTTTG
ATCACCTACTAACCAGTTAATCGCTGTGTCGCTGCGCTGGAAATCTTGACTAG
GGAAGGTGGTTTTACGCCTAAGGATGGCGTCATGGGCTTGTAGCCTCTGCGG
ATTAGGGACCTACCGACACAAGTATTTTGACCACAATGTGCGATTACCGTAA
GTAGCTTTAAGGCCTAATCGGTGACAGCCTAAAAGGTGCTTTTGTCGTGCGTC
AGTGTGACTCCAGCGAGCTGAGGACGTAATCCGGTTGAAGGCCTCGAACGAT

CTTTTGGTGGGTAACCCACTCTCTTTCCGTGCCATATGGATGACAACGCTTGT
AATCTACACCGACCGTTTTAAGGTCCGTGCGTGACAGCTTCAGGAGAGTGAT
GGTAGGTCCCAAAGCAGCCTCATAGACTGCTTTTCTCCTGGAGCCAGCGCTTT
TTTGCCTAGGCTCGTTACTCGAGTAATACCCAGGCTTTTTTCATGTGGTATTA
ACTCTTTTGGAATAACCTACGTCCCAGTGCAAAGTAACGAGCCAGGCATCGC
TCATGCCTAGCGCAGGTACTTTTGGAAACTTAGTGACCGTTTTTTGAACGACT
AACTCCGTGCTACATGCTTGCTGTCATGATTGCTGCTACAACGACGGCAAAA
GACATCGCACACATGTACTGCTGCACACCTCCTCCTTTTTTCTAAAAGGTGTG
TAGCTTTTACATGTGTAGCGGTTTAGCAGGACGTAGAGGCCCATTCTCACGTC
GTGGGATCCGGGCGTTGATTTTGTCTAACTCCGTCGACTTTTTTCAGGGCGGA
GATGGTGCAAGAATCCCTCGTGGTTTTTTGTTGGAGGGATGAGTCTTTTGGCA
TCTCCCAGGTGTTGTCGTGAATCTGTAATCGCCCGTACGAGTAAGTCTGCGAT
TGATCCTTTTGTCTGAAGTTCAGGGTGAT

23 crossing number 3-square knotted DNA after hierarchical design
ATCACCCTTGGTCTCAGACCAACTCGCCAATGCGACAAGCTGTCTCTGGAAA
ACGCACATACGTTGGAGTGGATGGAGATTCGACTCCCATGACCAACTTTTTTC
CACGTCATGGTCTTGTTTTCACCATACGTCCCCTGTTTTTTGTCGAGACGTTTA
GACGCTACATGCTTGCTGTCATGATTGCTGCTACAACGACGGCAAAAGACAT
CGCACACATGTACTGCTACACCGTGTTTAGTTTTTTGGAGGCACGGTGCAGCT
TTTAGAACTGAAGCGGTTTAGCAGGACGTAGAGGCCCATTCTCACGTCGTGG
GATCCGGGCGTTGATTTTACGGAGAGATCCGTTCTTTTTTCGGTCGATCTGTTT
CCGTACCTGCGCTTGGCCGATGTGATGCCTGGCTCGTTACTTTGCACTCTCCG
ACAGGTTATTCCGAGTTCGGTTTACATGTTTTTTGCCTGAAACCGACTCGTTTT
AGTAACACGAATAGGCTTTTTTGCGCTTTCGTCAGGAGTACTTCCAGGTGTAG
GTCCCTAATCCCGGCGTGCTACAAGCCCATGAACAGGGCCTTAGGCGTTGTT
GTCCGTGAGACGTACAAGGTATTACCACTCGACGAAAGTGACATATCCCCAG
AAATGTTGATCGTTCGAGCAGCTCAACCGGATTGGAGGATCAGCTCGCTGGA
GTTCTGGCGACGCACGACCTGCATTTAGTATCCCTGGATGCTTGCTGTCTTCC
ATGTGCAATGTAAAACCGCTGGAGTTGGGCAGTCTATGAACTGGCTTTGGGA
CCTACCATGCGGCTCCTGAAGCTGAACGACACGGACCTTTCGCGAGGTATGA
GGGTACCTTGCGCTCTTAGCCATGGTGCACCTCACACTTCACTCTCAGCTGAG
GCGGTGTAGATTGTCTCGGTTGTCATCCATATGGCACGGAAAGACAGCGTGT
TACCCACCGCACCTTTTAGGACAACACCGATTAGGCCTTAAAGCTACTTACGG
TATCTGGGCATTGTGGTCGAGGCAATGCTGGCTGACACGGACAACCTGGTCA
CTTGCGCTCCGTCGTCACGACTCTTGGGGCCACCTTCCCTTTGGAAGATTTCC
AGCGCAGCGACACAGCGATCCCGTGGTTAGTAGGTGATCGGTTCGCAGAGGC
CTGAGCTGTTCGCACGTCCGCTGCACCTCGTGACCACACTTGTTAGGAATCGA
ATGGGACGACTAATGAGCCTTTGAAACTGGTTGACGTCTCCAGAGGACGTGT
CTAAGGCTCTTTGGTGTCGTCAGGTAAGCGTTTGGCAGAGGCAAGAGAGCAA
ATTCGCCATAAGAGAGATCCCATGAGGTTGACGAGTTTTTTGCCTCCAACCTG
CAGCTTTTCAAGTTAAGTGAGACCTTTTTTCACAGCACTTCATCGCGGATCAA
TCGCTGTGTTACTCGTACGGGCGATTACAGATTCACTGCGACACCTGGGAGAT
GCCCGTAGACGTATGACTGTTTTTTCAGTGATACGTGATACTTTTATGTTGCG
TGCAAACCCGGGACGATTTGCCACAACCCCTTGCCAAAGCCCAACCAGTGTC
GGGTTTTCATATGACGTCAGTACTTTTTTCGTGTGACGTGGTTTCCGTAGGGT

CTGACTCAAGACGAGACTGGCAAATGGTACGAACTCAACGAGGAGTCCTACC
CATCTGCATCATGGCAACCTTTTTTCGGTTCCATGAAAGAGTTTTCTGAGTGA
CCAGTCTAGTTTTTCTAGACGAACTACTCAGCTCTTGCTCTTAACCGTTTTTTG
GTTGAAGAGCTGCAGTTTTATGGGTAGGAGAAGTGGTTGAGTTCGTACCATTT
GCCAGTCTCGTCTTCCTCCAGACCCTACGTTTTGAAACCTGCCAACACGTTTT
TTGTACTTGGCACATATGCCCGACACTGGAGTCGCTTTGGCAAGGGGTTGTGG
CAAATCGTTAACGGTTTGCACGCAACATGTATCCCCTCACACTGTTTTTTCAG
TCTGAGGGCTACGTTTTGGCATCTCCCAGGTGTTGTCGTGAATCTGTAATCGC
CCGTACGAGTAAGTCTGCGATTGATCCTTTTGCGATGGCCCACTGTGTTTTTT
GGTCTTGGGCAACTTGGCTGCGAAAGCGAGGCTTTTTTCTCGTGCTTTCCATG
GTTTTGATCTCTCTTCCCTGTAATTTGCTCTCTTGCACGCCGCAAACGCTTACC
TGAACCTGGCAAAGTTTTAGCCTTAGACACGTCCGAGTGAGACGTCAACCAG
TTTCAAAGGCTCATGCCACGTCCCATTCGTTTTATTCCTAACAGCCAGAGTCA
CGAGGTGCAGCTCCTCCGCGAACAGCTCGCTGCTCTGCGAACCTTTTGATCAC
CTACTAACCAGTTAATCGCTGTGTCGCTGCGCTGGAAATCTTGACTAGGGAA
GGTGGTTTTCCCCAAGAGTTCGTTCGACGGAGCGCACCGCACCAGGTTGTCC
GTGTCCAGTAGCATTGCCTCTTTTGACCACAATGTGCGATTACCGTAAGTAGC
TTTAAGGCCTAATCGGTGACAGCCTAAAAGGTGCTTTTGGTGGGTAACCCACT
CTCTTTCCGTGCCATATGGATGACAACGCTTGTAATCTACACCGCCTCTTTTA
GCTGAGAGTCTCCTCTGAGGTGCACCATGGCTAAGAGCGCAAGGTACGAGTA
TACCTCGCGATTTTAAGGTCCGTGCGTGACAGCTTCAGGAGAGTGATGGTAG
GTCCCAAAGCAGCCTCATAGACTGCTTTTCCAACTCCAGACGACGTACATTGC
ACATGGAAGACAGCAAGCATCCAGAGTGACTAAATGCAGTTTTGTCGTGCGT
CAGTGTGACTCCAGCGAGCTGAGGACGTAATCCGGTTGAAGGCCTCGAACGA
TCTTTTAACATTTCTGGGGATACGCAACTTTCGTCGAGTGGTAATACCTTGTA
CCACACACGGACAACATTTTACGCCTAAGGATGGCGTCATGGGCTTGTAGCC
TCTGCGGATTAGGGACCTACCGACACAAGTATTTTCTCCTGGAGCCAGCGCTT
TTTTGCCTAGGCTCGTTACTCGAGTAATACCCAGGCTTTTTTCATGTGGTATTA
ACTCTTTTGGAATAACCTGTCGGATCTGGCAAAGTAACGAGCCAGGCATCAC
ATCGTAGTAGCGCAGGTACTTTTGGAAACTTAGTGACCGTTTTTTGAACGACT
AACTCCGTTCAACGCCCGGCACTCACGACGTGAGAATGGGCCTCTACGTCCT
GCTCGTCGTCTTCAGTTCTGCTGCACACCTCCTCCTTTTTTCTAAAAGGTGTGT
AGCTTTTAGTACATGTGCCCAGAGTCTTTTGCCGTCGTTGTAGCAGCAATCAT
GTTGTCAAGCATGTAGCTTTTGTCTAACTCCGTCGACTTTTTTCAGGGCGGAG
ATGGTGCAAGAATCCCTCGTGGTTTTTTGTTGGAGGGATGAGTCTTTTGAATC
TCCATACGCTGCAACGTATGTGCGTTTTCCAGAGACACGAGACCGCATTGGC
GAGTTGTTTTGTCTGAAGTTCAGGGTGAT

57 crossing number 9-square knotted DNA before hierarchical design
CAACTCCTCGATTCCCGCTTGTTTGCACTTGTATGTACATAGTGTCAGATCGC
TTACGCTTGCGTGGCGATCATCTAGTCGTCGTTTTTTGTTCCACTAGATAGTAT
TTTCAGGCGTTGACTAGGCGCCCGAGGTATTTCAAGAAGACAACTGATTAAG
TGTGTTTTCAAGTCCAACTACTACTTTTTTGCGGAAGTTGTCCCTTCTCTACCG
TCTTGCTCAGTGTTCAGAGACTTTGCTGGTGAAGTGCCGCACCGCCTGCTGTT
AAGAGAGCTTTTTTGGGAGCTTAACGCAAATTTTCCACGTAAACTGTTGGTTT
TTTGACTCAGTTTAGGACTGAGTCAAGCATGGTCGGTCCCACAGAATCCGGA

AGCCCAGTCAGAAAACATGGTAGCGCTTCCCCTGGGTTTTTTCAGAAGGGAA
GCGCCATTTTGCATATTTACCACTCGTGACTCGTAGGGAGGACGCCTAGTGAA
GCGCGTCTCATTTTCTCGAGGAGTGAGGGCTTTTTTCCAGGCACTCTGTGTCG
TCCCGTTAAGGATGTGAGTGTCATGGCGAGCACCAGACAGAGGCCGTGAACG
CTTGAAGCGAGAGCCGTTTTTTGACATCTCGCTGGTGCTTTTCACCTAGCAAC
TATCGTTTTTTCATGAGTTGCCTCTGCTTGAGCATCAAGTCGTAATCGTCCATG
TTAGGTGCGCTTCTAGAAGACCGGTGTCCAGTATGGGTGTGATCTCCTGTCTT
GCCTGGTACCGCTTTCCAGGATCGGGTACGGGACTCGTGCCCGGAACATCAG
GGCAAATCGCGGTCTCATATAGGACTGTACCCACACACTTGTCTTCGCCGTTC
GGAACTTTCGGAGTATACACAGTCTTGTTGCGCTTTAACAGATCATCTGTCAT
AAGTAAGGGGTTGCACATATCCAAGATGGGATTGCAACGGTCAACCGGATAT
TACGTATTTTCCTGGGCTAGCGTCGGATTTCGGCTTCCTTGGGACTGAACAGG
GATCGTTTGGTGCTAGGCGCTGGCCTTTCACACGGGCGTGTGCAGCACAGAT
CATCTAATCATATGTAACTTGACCCCGTTTGCCTCATCAGTCCTCATGATGAG
TAAGTAGCCATCTTCAGTAAATCTTCGTCTAACATAGGGTACATACTCATCAT
GGACACTTTCTTCCGAATGCCTGCTACCCGCACCTCAACCGCCCTAAGACTAT
TGGTGCTTCTTCACTTCGTCTGAGTCAGCCTCTCCATCTGTCACTCCAAGGGA
TAGCGGACGACCCCGAGTGTCTTGAATTGCATCTACGAAAACGTTCGGACTA
CTTTTCTACGTCTGTTATCTCAGTCCTTGCCGACGTCGTAGGTCGTGATAACGT
CCAAGTCGGGTTGCGACCAAGGCCAGACGGAAGGTGGATTAGTTGTTACTTC
GCCAGTGAGAGTTTGCCTAGCTTGGATCACTGACGTCTGATGTTAGCGTAATC
TAGATCACTGGGGTTCTGCCACAGTCCGGTGAGTACAGCACAGATCGTATAC
ATCACGAACGTTTGTCCAGTCGCGGAACACTGAAATGAACTAACGCTGACAT
GTATGACAACCAACATGATTACACACGCTTGTGAGCGAGTCTCTGCATGAGG
AGTGCTCCACAGTGAGTGACTACGCTGCCAGCTGCAGCGCGCGTTTGTTTCGG
GTCCCTTCAGCATCGAACTGATCCGCTAAACGTCTTCACAGGCAAGACGTCG
CGAATAGCGGAACGATGCAGGTATCTTAGTCATGCGCAACCAACCACCACAG
GTGTGCTACTACGATTCGTCCGTCGGCATTTAACGCTCCGCCGTTGGATCGAT
AAATGGTTTCTGGAATTATATAGCCGGTAGATCGGCCGAGACACTTACGTAT
AATGAGCCCACTTATCCTCTTCTTGTGAACACTGCTTGCTATACTCTTGCACA
CCTCGGTCGCAGACGGTAAAGACTACTGAATACGGCGATAGCGTATGTGCGC
ATGACGCGCGGTGAACACATGGTGCCAAACACTTTCTTCGTGACCCGAATTG
AATAATCGCTTATACAACCTGAAAGTGCCGTACGCATTCGCCGTGGGCTAAA
GCGCGATCAAGTCTCACCCTTGGTAGTCGAGTTGTCGGGCGTAGCAGCTCTCT
GTCCTCGTCCCATTACTTTGCTTAGTGAATGGAAGACTGTGGTAATGCTCCTC
ACGAGGATAGTCAACTGACCGTCATGCAGCTGACGACGTTTAACGATCCTGC
ATCTCTCCCAAGATAGTCAGATCCGTAACGAGACTTGCAGGACTCGAATACC
CTCTAACTATCCGCACTAGAGTGCCGGAGGCAGTTCGCCCTTGTAGGATCGG
AGGTAGTCAGTTGATAGAAGGGCTGATGGCTCGTCCTCGAAGTTGAACTCCC
TCAGCCACACTTGGTTCCTACAGCAGTTGCATCTGAAGAATCTGTCGACACAG
ACATGCCTTCGGCGCTCGTCCCACTTATGCAAGCGCATTCGTCGAGGCAAGG
AGCTCACTTAGAGCGTCTGATGGTTTAGTACTGAGTGAGATATCGTCCCTTCT
AATGGACTTTGGTCCTGCGGTTATTTTGGGAGCGGCACATGACGCGCCAAGC
ACTCATACTTTTTTCGATCAGTGCTCGTACTTTTCTTATGCTTCGAGCCACGTT
GCCGCGCATATCAATTCTACCTAGGCATACCTGTTTTCTTAACAGCCACTGTG
TTTTTTGCGGCTGGCTGGTCAGGGCGACTCTGAGAATAGAAAGGTGCGTGTA

ATCATAGAGTGAGAGCGGGTGTCTGTGGTCTCCGATATCTTTTTTGAGCGCGG
AGAGTTTATTTTATGCCTCGTGGGGCTCTTTTTTGTAGCCCACGTCAGATTGCT
GACAGATCACGTTGATTTCGTTCCAGTAAAGTGAAATGAACTCTGACCGGGA
GTGCCAACGATACTTTTTTGTAATGTTGGCGCATATTTTAAGCGCGTGACTGC
ATATCAGATGAGATGTTAGGGAGGTGAGAAGTACTCACCTTTTGGCGTGCAC
GCCGTGGTTTTTTGATTAGCGTGGTCGGGTGGCCGGAATGGTATGCCTAAACA
CCAGACATCTGAACTGTCTGGTTCATAATGTGACACTTGTCTGGCTTTTTTCGT
GAACAAGTTTGTATTTTACTAGGGAGGATGGGCTTTTTGCCCACGGATCCTAG
TTACAATGATCGTCACGTTTTTTGCCAGCGATCAGTCACTTTTATTATGAACC
GTAATCTTCAGATGTCTGGTGTTTAGGAGCGTCATTCCGGCCATTTTCCCGAC
GGACTTAATCTTTTTTCCACGAGTCCCACGCCGGTGAGTACTTAAGCTCTCCC
TAACATCTCATCTGATTGTCCATCACGCGCTTTATGCAGTATGATTACTTTTTT
GTATCCATACTACTCCTTTTCGGTCAGAGTAGCCCACACTTTACTGGAACGAA
ATCATGACCATCTGTCAGCATTTTATCTGAAAGTTGCTACTTTTTTGAGCCAA
CTTAGGCATTAAACGACCGTCGCTCTTTTTTGATATACGGTCCCACATTTTGA
CACCCGCTTGTGCACTATGATTACACGCACCTTTCATCTGTCAGAGTCGCCTT
TTCTGACCTAGGCGCCGCTTTTTTCACAGGCCTAGTTAAGCAGGTATGCCTGA
CGTGAATTGATATGCGCGGCAACGACTGTAGAAGCATAAGGTACGTCTATCG
ATCGTTTTTTGTATGGATAGATGGCGTTTTCGTCATTCTTCGCTCCCAAAACCT
AACCAGGACCAAAACGACTTAGAAGGGACTTTTGATATCTCACCTCTGTCTA
AACCATCAGACGCTCTAAACATCCTCCTTGCCTCTTTTGACGAATGCGACGAA
GTAAGTGGGACGAGCGCCGAAGAGGGCTCTGTGTCGACTTTTAGATTCCCTG
GATGCAACTGCCAGGCAAACCAAGTGTTCACAAGGGAGTTCAATTTTCTTCG
AGGACTACAGTTCAGCCCTTCTATCAACTGACACGTCCCGATCCTACATTTTA
GGGCGTGTGGCCTCCGGCACAGTTCCGCGGATAGTTGACAAGTATTCGAGTC
TTTTCTGCAACAGCCGTTACGGATCAACGATTCTTGGGAGATCCCAAGGATCG
TTAATTTTACGTCGTCAGTGGACAGACGGTCAGTTGACTATCCTCAGCTTGAG
CATTACCATTTTCAGTCTTCCAGACTGGAAGCAAAGTAATGGGACGAGGGAT
CTGAGCTGCTACGTTTTCCCGACGTCACGACTACCAAGATTAGAACTTGATCG
CTGCACAGCCCACGGCGTTTTAATGCGTACGTGAGACTCAGGTTGTATAAGC
GATTATGGGCATCGGGTCACGATTTTAGAAAGCAGGTGGCACCATGTAAGTG
TCGCGCGTCATTATGTCATACGCTATCTTTTGCCGTAACTGGTAGTCTTTACGT
AGAACGACCGAGGTAACGTAGAGTATAGCATTTTAGCAGTGTTCTGACTGAG
AGGATAAGTGGGCTCATTACGACCAAGTGTCTCGGTTTTCCGATCTACCACTT
CAATAATTCCAGAAACCATTTATGAGCACAACGGCGGAGTTTTCGTTAAGCC
TCGACGGACGAACGACTTTAGCACACCTACGACGGTTGGTTGCGTTTTCATGA
CTAAGTACTGAGCATCGTTCCGCTATTCGCGAGAGGATGCCTGTGAAGTTTTA
CGTTTCTGTGATCAGTTCGAAGTGATAGGGACCCGACTAACACGCGCGCTGC
TTTTAGCTGGCAGCCGTAAGACTCACTGTGGAGCACTCCTCCAAGTGAGACTC
GCTCTTTTACAAGCGTGTAGACAGATGTTGGTTGTCATACATGTCCATACTAG
TTCATTTCTTTTAGTGTTCCGCTTCACTACAAACGTTCGTGATGTATACACAGA
GTGCTGTACTCTTTTACCGGAAGCGGGCAGAACCCCTGCTGACTAGATTACG
AACAAATCAGACGTCATTTTGTGATCCAAGGTCCTAAAACTCTCACTGGCGA
AGTAATGGTCAATCCACCTTCTTTTCGTCTGATGCTGGTCGCAACCTCGTAGG
GACGTTATCGTGGTCTACGACGTCGTTTTGCAAGGTTCAAGATAACAGACCGT
CTGAAGTAGTCCGGTGCATTTCGTAGATGTTTTCAATTCAAGAATACGTGGGT

CGTCCGCTATCCCTTGGCAACGCAGATGGAGAGTTTTGCTGACTCAGCTTGCA
TGAAGAAGCACCAATAGTCTTGCATGGGTTGAGGTGCTTTTGGGTAGTGTTCA
TTCGGAAGAGTTCACCCATGATGAGGCGCAACCCTATGTTATTTTGACGAAG
ATTATACCTAGATGGCTACTTACTCATCATCGTCTCTGATGAGGCATTTTAAC
GGGAACTAGTTACATATGGGTGAGTGATCTGTGCGCTTTACGCCCGTGTGTTT
TAAAGGCGTCTGCCTAGCACCATGACTACCCTGTTCAGGATGCAGGAAGCCG
AATTTTATCCGACGCTTCATTTGGAAAATACGTAATATCCGGTACGTGGTTGC
AATCCCTTTTATCTTGGATACTCACTACCCCTTACTTATGACAGATGTATTCTT
AAAGCGCAATTTTCAAGACAACTTATACTCCGAATCTAGTGAACGGCGAAAG
AGGGTGTGTGGGTATTTTCAGTCCTATAGCACTTCGCGATTTGCCCTGATGTT
CCTCAATCGAGTCCCGTATTTTCCCGATTTCAGAAAGCGGTACTGTAGGAGAC
AGGAGAGGCTGCCCATACTGGATTTTCACCGGGTGCCTAGAAGCGCATAACC
GATGGACGATTGTCCATTGATGCTCAATTTTGCAGAGTTTCTTCATGTTTTTTC
GATAAGAAATAGGTGGCACCGTGGTCATGTCTTTTTTCGGCTGACCACTCAAG
TTTTCGTTCACGGCTCAGTACTGGTGCTCGCCATGACACTCGTGAGCTTAACG
GGACTTTTGACACATCCGTCCTGGTTTTTTGCCCTACGGACTCGAGTGAGACG
CGCTCGTTGAGGCGTCCTCCCTACGAGTCAACGTATGTAAATATGCTGGCGA
ACAAGTTCTGTTTTTTCCCAGCTTGTTCGCTATTTTCCATGTTTTCACAAGAGG
CTTCCGGATTCTGTGGGACTACGTATGCTTGACTCTTTTAGTCCTGAGGAGAG
TCTTTTTTCCAACTCCTCACGTGGTTTGCTACATCCTCCCTTTTTTGCTCTGATG
TAAGCAGTTTTGCGGTGCGGCGGCTATCCAGCAAAGTCTCTGAACACTCGAT
CAGACGGTAGAGTTTTAAGGGAGCCTATCCGCTTTTTTGTAGTTAGGCGACTT
GCACACTTAATCGACCATCTTCTTGAAATACCTCGGGCTAGGACTCAACGCCT
GTACTACTGCAGGGAACTTTTTTCGACGCTGCAGTGATCTTTTGCCACGCAAG
GTAGTCCGATCTGACACTATGTACATAATGCAGCAAACAAGCGTTTTGGAAT
CATCCGGTTGG

57 crossing number 9-square knotted DNA after hierarchical design
ATCACGACGCTGTTTCACGGTTAACTCCTCACGCTCCACAGACCAGTACTTCC
GACTTTTCAACCTTGTACCACTAGGAGTGTTTCCTCGAGTTAACCTTTTTTCTC
CAACTCGATGGTATTTTAAGATTGGTGTCTGGCACATAGCTGCTCCTAGTACC
AAACCTTAGATTCCTACGTTCTATAGGTTTTCAGAGGCCAAGATCGGTTTTTT
GGGAGCTTGGCACCACGCTCATGTGTCGGGATCATAGGCTGACGATTACTCG
GTACTTGCGATCATGACATAGAGATTGTTGTGATTCGCTGTCCTTTTTTGTTGG
GCGAATACAGGTTTTATACCACTGACGAAGGTTTTTTCGTGTGTCAGGCATAG
GTCGTTCGTAGGGTCCCCTATGCGTTGATAGAATTTGGTGTTCGGAACGTCAG
AACAATTCGGGAGCAGCGTCATGGACTTTTTTCGAAGTGACGCATGACTTTTG
CGTACTGTTCCCTCGCAAATATGCCAGGAGTGTGAACACATCCTGGTCACCCT
CAGGGACCATTTTACGATGTCCTCAATTCTTTTTTGTCCCGAGGAGCTCCCCTT
GCTCAATGGTGACGAAAGTCATACCGTCCAGAGCCGTGTGGATGGCTACGGT
CAGAGGAGGGATCTCTGTCCTGGTATGGGTGGAAGTCCTCGTGAGAGTTGGG
TGCGCGTACATTGCCAGACTTCGGCAAGTTTAGCCTTAACTCGTCAGGTGCCA
GACATCCTCTATTGTCCGACAACTGTAGTCTCAAAGGCGTCTGGCTGCACGGC
TTATTACGACGACTCCGTCAATGGACTGCCTTGACAGTCGGTGGCATAGCGGT
GTTATGAGTCAGCGTGAGGTTACTACGATACTACGAGGGACAGATGTTCTCT
GTACATGTGCAAGTGGGTGCCATTTAAGCTAGTAGCAGAGTGCGATGTGCAC

TGTGGACTTCTTCATGCTTGGACTTGTAAACCGAGAGCTCACGACACCATTCA
ATCGGCGACAGAGCACACCGTCGAATGCATAGGACCTTGGCTTTTGTATCCT
ACGGTGCATAAATGCCAGAGATGATGTAGCTCTCCGATATGCGATGTGACAC
CGTACCAGCACGTACGTATCCCATGTACGGACCTTGATCGCGCGCATAACCG
TCCGCCCACTATGTCGGCTGCTGCTACTCCCTCGATTATGTACCCTAAAAGGC
ACGTAGCGTGAAGGGGCATCATTTGGTCCACAATGTGCTAATGTTTGAAGCC
ATAGCAGGGCGGGTGTATGTATGCCGTGGTGAAAGCGTCACGCTGGGTAGCA
GCGATGGCCACCAAGCAGCGTGCATCAGGTCCAGAATAGCCTCGCAAAGCCA
GAATAGACACACCAAGTCCATGTCCAGTCTCGCAAAAGTAGTGAAGGCTTTT
GCTGGGTTTCGACCTTAGTTACTGGCAGCATGGATAGCACATGACGCCAACG
TGTGAAAGTACCAATGTCGACTGCGCGAGAGTTAGACTGAACGGCAGTACGT
GCTATCTCCTGCAGCGGTGATTCAATCTCGGAGGAGTAGGCAGAGCGTCGAG
GAATAGTCACTGGCGATAATCTTGTATTCGAGCGCTGCCGCATGCGAATTAGT
CACCGTGGGTGTATCTACGTTGGCTGCAGCTCGCACGTCGGCTGACTCCGTAC
CGCTCTTCCGAACTATCGACCATAACCTGCCGGCGAACTCCTTTGCTAGGCTC
CGGAAGCATAGACAATAGCTTCGAGTACCCATGGCCTTGTAGCTGAAAGATG
ACTTGCCACAATTGGAGGCTCGTTCCAATCTGTCTGCGATTCAAACCTCTTCC
CAAATCTATACGGTTACCTCGCAGGGCTCCTCATTTCCATGATTCATTCTACC
CGCTAACCGTCCTTGCCTGGAAATAACCTGTCCGTAGCAGGTAGACCGCTTGC
CATCGTCAGCGTCCTCCGTGCGGTAAACTTGTCACTGAGGGTTACCTTTGGTC
AAGTTTCTGACCGATCAGTGACACCCAGACCTTTCCACACCCCGAAGGCTAG
GCAAGACTGTGGTATGGAAGTGTGGGTGTCCCTGCCTACGCCTCATGTGGAC
GCACTCATTGACCGACTCGTGGTGCTCTACATGTTCCACAGCAACGGAAGGG
GACTCGATGGTGCGTGCTAGAGAGTTCATGTACGGAATCCATGTCTATCCTAG
GTCGATTAGGCGTTGACGGTTGCTCGGGAGTCCTCAAGTTCTATCTATATCGG
GGCATCTGAGATACGGTGTCTATCCATATACTCCGGTACCGATTAACAGGTTC
GATATGCCCTCAAGACTGGGTTGTACAGAGCAGGCTGGTCCATCTAGGCTAG
TTATTGCGTCCGGATGGCTGCAGGCCACGCATGCCTACGCTTAGTATGCCTGC
AGTCTAAATGTTTTGTCCGGTCAGTGACACTTGACGAAGAATGAGTGGATTGT
GCTATTAACACGTCGGCTACCTCACATTCTGTCTACCCTTTACTGCCTCGAGC
TCCGAGCCGGAACGACTACACAGTTTCTGAAAACTTCGAAACTCCATTTGAA
ACTGCGAGACGCTTGCACGTCTACGCATGGCTGTTCGTAACGGAGTGACCGT
CACACTACAGGGATACAGCAAGCCATTCGCGTTGCCAACAGCGTCTAACATG
TATTAGAGGAGTCGGATTTCTTATGTGTCGCGTGCGGACTCTCGATCTCGTTC
CACTCTCTGGAAAACATTTGTCTTAGGGGTGTTGACCCGTTCGGTTGTCATTG
AAATGACCCGACTCGCACTAACTGCGTATTGGTTTCTCCAAAATGTTGGGGTC
ACTCCAAGGTGACGTCTCGTGCCGTGTTGCGCACCATTTCCTTCACGTTAGTA
CCACTTTCTCTGGGGTAGCTGAGGGTACAATAGGTTTTTTGTAGCTGTACCAT
ACGTTTTCATGACTGTGAATGTCTTTTTTCCTGATCACATGACGGTGCTGAAC
CGAGCCTTCATCAACGTCCTGAGTACTGCCTACAACAAGTTCTGGTCTGCTGG
ATTACCTGGGAGAGTAGGTTTTTTGCCACTCTCCCTCGACTTTTGTTATCTTGA
CTAGGAAGCGAACGCCTGGGTTGGTCGTCGATAGCACCACGCTACCAGACCT
CTTTTGAGGTAGCTCCTACCCTTTTTTGAACTGGAGCCAATTGCAACATGCTT
CCGACTCATGGTCATCATTGATGGGTCATCAATACGTGTTGGACAGTAGATGC
GTACCTGCAGCACGAGTTTTTTGCAAGGCTGCAGACACTTTTATTCCCATCTC
AGGACTTTTTTGTACCGAGATAATTGTCTCATTGGTGCCACCATCACCGTCCA

CCAATGGCCCAGATGCGATTCCAAAGCTGTGCTGTCCGACTATTGTCGTACAC
TTTTTTATACTCGACAAGGCAGTTTTCGAACGGAACTCAGGGTCGGAACCATT
ATCCGTGCGGATACACGCAATGTGACACTACCAGGATTTTCCTCTACCTAGCC
TAGTTTTTTCCCTTCTAGGTGACACGGCATGTGACTGAGGCAAAAGCGGTTTC
TGCGATGGCTAATTTCGTCGAAGCCGATCAACTATTTTCCAGAGAGGTACCTT
TTTTGTGAGCTCTCTCTTGCTTTTGAGACTACAGCCTCTAGTTTTTCTAGAGAT
CCCAGTCTCGCAAGCATCTCCTCACTTTTTTGGTACGAGATGGGAAATTTTAT
AGTTGATCGGCTTCACTCAAATTAGCCATCGCAGAAACCGCTTTTGAGCAAGT
CACATGCCTTTTGTGTCAAGCCAAAGGGTTTTTTCTAGGTGGCTTAGAGGTCC
TGGTAGTGCGTGATTGCGTGTATCCGCACGGATAATGGTTGTGCCCCTGAGTT
CCGTTCGCTGCCCAGGATAGTATTTTTTTGTGTAATCCTGTAGTCTTTTGGACA
GCACACGTTCAGAATCGCATCTGGGCCATTGGTGGACGGTGATTCACGCACC
AATGAGTTTTACAATTGACGTGGTACTTTTTTGTCCTACGTCGGGAATGTGTC
CGTCTACTTGCTTTTTTCTCGTTAGACGGGTACTTTTGCATCTACTGCATGCTA
CGTATTGATGACCCATCAATGATGACCATGACTTCGAAGCATGTTGTTTTCAA
TTGAGCAGAGTTCTTTTTTGGGTACTGCTTACCTCGAGGTCTGGTAGTCGGGT
GCTATCGACGACCAACCCAGGCGTTCGCTGTAGCATCAAGATAACGTCGACA
TCTGGTGGCTTTTTTCCTACCAGATGAGGTATTTTATCCAGCAGAGTACGCCT
TGTTGTAGGCAGTACTCAGGACGTTGATGACCAGTCGGTTCAGCATTTTCCGT
CACTCACTCAGGTTTTTTGACATGTGAGGTCATGCGTATATACTGGCTACTTT
TTTCCTATCAGTATCTCAGTTTTCTACCCTGACGAAAGTGGTACTAACGTCTCT
GAAATGGTGCGCAACACTCACCGAGACGTCACTTTTCTTGGACAAGCCCCAA
CATTTTGGAGATCGTAATACGCAGTTAGTGCGCCAGGGGTCATTTCATTTTAT
GACAACCGGCTTCATCAACACCCCTAAGACAAATGTTTTCATGCCCGTGGAA
CGAGATCGATTTTGAGTCCCAGTGCGACACATAAGAAATCACTATCCTCTAAT
ACATGTTATTGCCTGTTGGCAACTTTTGCGAATGGCTCTGACGATCCCTGTAG
TGTGACGGTCACTCCGTTACGAGACCCCATGCGTAGATTTTCGTGCAAGCGGT
CATGAGTTTCAAATGGAGTTTCGAAGTTTTCAGAAATCCCGTAGTCGTTCCTT
TTGGCTCGTCTGTCGAGGCAGTAAAGGGTTCGTAGAATGTGAGGTAGCCGGT
CCGTTAATAGCACTTTTAATCCAGGGTTTCTTCGTCAAGTGTCAGCAGCCGGA
CAAAACATTTAGGGACCAGGCATACTATTTTAGCGTAGGCAACACCCGCCTG
CAGCCATCCGGACGCAATAACGCTCGAAGATGGACCAGCCTGTTTTCTCTGT
ACAATGGCCACTTGAGGGCATCAGCAACCTGTTAATCGGTACGCATGTATAT
GGATATTTTGACACCGTATTCGGACTGCCCCGATATAGATAGAACTTGAGGG
TTAAGGAGCAACCGTCAACGTTTTCCTAATCGACTGCTACTAGACATGGATTC
CGTACATGAACTCTCTAGCCGACACCATCGAGTCTTTTCCCTTCCGTTATGGA
CGAACATGTAGAGCACTGGCTTTCGGTCAATGATCTGGTCCACATGAGTTTTG
CGTAGGCAGTCCTCGCCACACTTCCATACCCTCCGATTGCCTAGCCTCGCTGG
TGTGGAAAGTTTTGTCTGGGTGTCACTGAGCACTCAGAAACTTGACCAAAGG
TAACCCTCACACGCAAGTTTACCGTTTTCACGGAGGACTGGTCGGATGGCAA
GCGGTCTGGTACGTACGGACAGGTTATTCGAGCTCAAGGTTTTACGGTTAGC
GCCGTACATGAATCATGGAAATGAGGAGCCCTGGTCACAAACCGTATAGATT
TGTTTTGGAAGAGGTTTGAATCAGGTACAGATTGGAACGAGCCTCCAATTGT
GGGCGTTCATCTTTCAGTTTTCTACAAGGCCATGGGTGACGGAAGCTATTGTC
TATGCTTCCGGAGCCTCCTCAAGGAGTTCGCTTTTCGGCAGGTTAGCTGACAT
AGTTCGGAAGAGCACCTGCGAGTCAGCCGACGTGTCCAGGGCAGCTTTTCAA

CGTAGATTGCGTGACGGTGACTAATTCGCATGCGGCAGCTAGCCTATACAAG
ATTATCGCTTTTCAGTGACTATGGACACACGCTCTGCCTACTCACAGTCGATT
GAATCACTCGGGCAGGAGATAGTTTTCACGTACTGCGCTTTGGTCTAACTCTC
GCGCAGTCGACATTGGTACTTGGTGACGTTGGCGTCTTTTATGTGCTATCTCC
AACGCCAGTAACTAAGGTCGAAACCCAGCAAAAGCGTCGACTACTTTTGCTT
TTGAGACTGGACGCTGTGTTGGTGTGTCTATTCCACGAGTGCGAGGCTATGTG
CGACCTGATGCATTTTCGCTGCTTGGCCCAGTTCGCTGCTACCATCGGTGACG
CTTTCACCACGCGGAACATACACCCGTTTTCCCTGCTATGAACGGGAACATTA
GCACATTGTGGACCAAATGCAGAGACTTCACGCTACGTGCTTTTCTTTTACTC
AACATAATCGAGGGAGTACTGACAGCCGACATAGTGGGCACTGGGTTATGCG
CGTTTTCGATCAAGGTGGTAGAATGGGATACGTACGTGCTGGTACGGTCGAG
GTTCGCATATCGGAGAGTTTTCTACATCATCGATCAGATTTATGCACCGTAGG
ATACAAAAGCCAAGGTGAGCTGCATTCGACGTTTTGTGTGCGAGCTCGCCGA
TTGAATGGTGAGACGAGCTCTCGGTTTACAAACGTAAGCATGAAGATTTTAG
TCCAGCACGCACATCGCACTCTGCTCGACGCTTAAATGGCACCCACGACGAC
ATGTACAGATTTTGAACATCTGTTGTCGATAGTATCGTAGTAACCTCACGCTG
ACTCATAAGTTGGCTATGCCACCTTTTGACTGTGTGAGCAGTCCATTGACGGA
GAACCCGTAATAAGCCGTGCAGAGTCACGCCTTTGAGTTTTACTACAGTTGCT
CAGAAATAGAGGATGTCTGGCACCTGACGAACTCCCGCTAAACTTGCCGAAT
TTTGTCTGGCAATCCAGAAGCACCCAACTCTCACGAGGACTTCCACCCATAA
GGCGACAGAGATCCTTTTCTCCTCCAGACGTAGCCATCCACACGGGAAGGGA
CGGTATGACTTTCGGGCACATTGAGCAAGTTTTGGGAGCAGCGTGGGACTTTT
TTGAATTACGCTCATCGTTGGTCCCTGAGCAACACCAGGATGTGTTCACACTC
CTGGCATATTTGTCGACAAACAGTACGCGTCATCACATGCTTCGTTTTTTGTC
CACATGTGTGCTCTTTTCCGAATTGTTTGCTGTTTCCGAACACCAAATTCTATC
AACGCATAGGGACAGCTACGAACGACTTTTCTATGCAGTCGACACGTTTTTTC
CTTCCGACTTGGTATCCTGTTCGTCACCAACTTTTTTGGACATGACGACACAA
TTTTCAATCTCTATTCTCGCATCGCAAGTACCGAGTAATCGTCAGCCTATGAC
TGTGACACATGAGCTTTTGTGGTGGTCTCCTCCCTTTTTTCCGATGAGACCCTC
TGCCTATAGAACGGCTCAATCTAAGGTTTGGTACTAGGAGCAGCTATGTCTG
ATCCACCAATCTTTACCACACCAGTGGAGTTTTTTGGTTACTGGTGGGAAATT
TTCACTCCTAGTGGTACAGCAGTGAAAAGTCGGAAGTACTGGTCTGTGGACA
AGGAGGAGTTAACTTTTCGTGAAGGGATGTCGTGAT

67 crossing number hexagonal knotted DNA
ATCCAGGGAGTGGCGTACCGACACAAGAGTACGCCCCTAATGCATGGAGCCT
CGTCTAGGATATTTTCTTCCCCAGGCCTATTCACAGTTTTTTGGTCTCATAGGC
AGCTAGCACTGCTAGTGGGATGAGCTGGACACACTTGTGGTGAGGCAGTGCG
TCAACCTAGGCTTACGGGACTACCTACCTCCCTGGGAGGCTGTCTTCACGGAT
GCTGCTTTGGTCTAAGCCTTTTTTCCTGTCGACCAATGAATCGCACTTGGCCA
TGCTTTCTGCATTTGGGCTAGGATAAGGACCATGCTCGTGCACGTACAGAGA
AGACTCTTTTTTGTCCATTCTCTTAGTCCGGTATGAGCTTATGGACGAAGCCC
TAGGATCTGTAAGGAGCATCGAGTAAATCGTTCGTGTGTGGCATAGCGACGT
CAAGCAGGTGTACCTGTTCGAGTTTTCCTGGAAGAGTTCCCGTTTTTTGGACC
ACTCTTATGGCGCAACCATCGAAATGGGGCTGTAATCGCCTGGATGGAGCGA
TTCCCTTTAGGACCGTCCACTGCAGTGGACTTTTTTCGAGTCTGCAGTCCGAC

AAAGGCAGGTCTGTTGGTCTTCGGTATGAGCATCCGCAACCACATGTGCCCA
GAAAGTAGGATCACTTCTCTGCATGTACGTCTCAGCCAAGAGGAAGAAGCCA
GAGACACTCATGTCTTTTTTGTATGCAGTGTCAACGACAAGCGGTTCATTGGG
ATTGAGTGTCTATGATTCGTTCATCTAGGCTCGACCGAGAGTAAGCCAGATGC
CTTTTTTCTTAGGTGGCTTTGTCGCAAATCACGGAACCAAAGGATGCATGATT
AGCGGAGACTCTCTTCCGATTTGCAATCGGAGGTTCTGATCGTACCACATGCC
GACTTACAACCATGGTAACATGTTTCTCGCCAGGTAGATCTTCGTACTTATCC
CGATAGAGTTCTTCGTTTGAGCCTCTCATGAGTTCTACACTCTTGTCACTTCGG
TACAGAAGTCGTGGATACCTGATTGGTTAAGATCTTCCACATGGCACGACAA
CAGAGGCTTGCGTGCCTGGAATCGTTAGTGATATTCCTACATTGCCCCATTCC
TCCGCTAAGGGTACGGTACACCAGTAGGCATGTCAACTCACACACACTGGAG
TTTGGAACAACGAGAAATTGTAGGCAGCCATGGTAACGTTTATAATGTAACA
TTTCGGAGGAACAGACAGCTCATCGCCTTCACACGGTACCATGTCCTCAATCC
TGGGGACATCCTGACATGCTAGGATAAATCGGGCTCTACGACGAACTTGGCC
TAGGTTTATAACGCGTCTATATATCTCGGAAAATGAGATACCTAGGGGACCC
TACACTTTCTCTACACCAAGCATCTGGCCCTACATGAGTTTCACATGGGTTGG
AATCTAATGGTTGGCAATCGCAGAACGTGCCTTTAACCTGGGAGTAACAGAC
CTACGATTAGCCATTATCCCCTAATCTGCCAACCATTTGACACGCTAGGGATA
GATGCTTAGCCCGAATCTTGCACTTGTGTTTCCATATGGCGGATCTTCGAAAC
TCATGGCCACCTTTCCCAACGCATTTGGATCTTTGTGCAACTCGAAGGGACAA
AGGTTCCTATCAATCAGTGTAAACCGGTCGCTCATTTCCGCCTGGTGCTCGTG
CGTTGCTTACCTAATACACTCTAAGGAATTTGGAACAAGCTGGTGACGGACG
GCTGAGGTCGTCCTGTTTCGGGTAACTGCACACTAGCGTCGTATCGTAGGGTA
AGTGGCTCAGGGGACGCATTTGCTGACGGTCAGAATATCGCGTTTAATCCAG
TTGTCTCGACGATTTGCAGGACCTTAATACGCTGCATGTCAACGGCTACGTTG
ATAGTGCTTCTTACGTTTCACTCTCCAGTAGCTACAGCTCGTGAATCCCCAAC
ACTGAGCATTTAACGCAGACCGTTAGATGTACTCACACAGTCCAACGCTTCA
CTAGAAAACAATTTTCTAGGTAACGCGCGAGACTCTAATCTTGTCCACACGAT
CGGATTAATGCCGGAACTGCTGATACTAGCCGTCCCTCTCGATGTGTGACTTC
GGACGATCCTTATTGGATGCTATGGACTCCGACTGCGTAGGATAGAAGATAC
CGGCTACATCCAGAGCCATATGAAGTGTCCGTTGTGTCGGTTACGGTGTCCTA
ACGGTCTTGGGACCCGTATCCTGCTTGCAACACAGCTTCTTTCAAGGTTGCTC
GTTTGAGTGGTCTCCCCAGGGTTATCAAGACAGTGGCCATACTAGCGTTCCAC
TCGGTAAAATCCGACCTCGGACACTCAGCCTGAGACGCAGAGAGAACTAGGT
CGGCTGAAGCCTTTGGTGAGTAACGACCCTTACTCGGAGCGTGTGTTCGAAG
GAAAGTCATTGACAGAAGGCACGTTTTCTCAGGACCTAGTGTTAGGTTACGG
GCTATCACATGAACTGGCTTATTGAATCTTTCACCATACAACAGATGTGTATG
TGCATCCTATCTAAAGCACTGTTTGTCCAAGATGAGGATCTAGTATCGGTTCC
TCCTCACACACTGGGACACTTCTGTTTGACCCAGACTGGTCCGAATGCTACAT
GTAAGCTCTTCATAGTCTTTTCCAGCGGCACTACTGTAGGAGCCCAGAGTCAC
AACCGCCACATGGACACCCGCTGTTGTACTCCACGATTGGCATGGTAAGTGA
AGTTCTAGGTCAGAGGGTCCTATTCACGTGCGATACTAAGTGATACGATAGC
CATCGTGACTTGTTTGTGACTACCAACCACCACGTCCTACTCTTGAGGAAAGG
ATCACTTTCCTACCGTAGCGATCTACTACCCAGACCACACCGAAGGCAAGAT
CCCTTCTTGTTTGATTGGCTAGCCATACTACGTGAAGTTGTGCGAAAGTGGGA
CGTCTACCTACCGTCTCATATTGTATCATAACCGAGCCTGACGTTCTGTGGTA

GACACTTCGAAACCTTGCCTTTTTTGGTTGTGTTTCTCGTCTGTCGACCATCTA
CGAAGACTTCGTCGCATGCTGTTCAAGATGAAAGTCGGAACGGACAACCACA
GTGTCCTTTTTTCTGTGATGTGGTAGTACGTAGCTGCAGCTGTCAACTATCGG
ATTCTAAGCGCGAGTGTGCTGCTGAGGTAGACGTTGACACCACCCGCCACAC
CATAGTCTCTGGATGAATGACCCCGTGGGAAGTGTGACGTGGTTTTTTCTCTT
GCACACTAGCCGTATAGCCCAAATCCTACAGTCTTTGTCTGGCTATATGCCAA
TTGCGACGAATGCTATCACAACATGGATGTTTTTTGAGTGGTGTTGGATGCGT
GAACATTTCTACACACGTAGAGAAGGACGTGCACGGAGCAGTCTTGCACTGG
GCACATGTGTGTAGTCTTGCTATCCAACACGGGATGACTTGTGCTGCATCGTG
GCACTCGTCTAGTTTTTCTAGACTCCACCCACGATGCAGTTTCACAAGAGCCA
CCGTGTTGGATAGCAAGACTAAGTGTGTGTGCTTTCCAGTGCAAGTGATGGC
CGTGCACGTCCTTCTCTACGGACCAAGAAATGTTCATTTCGCATCATGACCCA
CTCTTTTTTCATCCAGTCATTGATAGCATTCTTTGTCGCACGACACATATAGCC
AGACAAAGACTGACCACGTTGGGTTTCTATACGGCTTTCCACCAAGAGTTTTT
TCCACGTGTGGAATCCCATTTCGGGGTCATTGCAAACGAGACTATGGTGTGG
CGGGTGTCACAAACGTCTACCTTTTCAGCAGGTTGGTCGCGCTTAGAATCCGA
TAGTAAGATCCTGCATTTGCTACGTACTTGAGACTCACAGTTTTTTGGACACG
TCTCATGTCCTTTGTTCCGACTTGTGCAATGAACAGCATGCGACGAAGTCCCT
AGAGATGGTCGACTTTAGACGACCTCTACAACCTTTTTTGGCAAGAGAGGGA
AGTGTCTACTTTCACAGATTAGGAGGCTCGGTTATGATACAATACTGTTAGGT
AGTTTGTAGACGTCCAGAGAGCGCACAACTTCCATGAGTATGTTGGTCCAAT
CCAAGAAGGGATAGATGCTTCGGTGTGGTCTGGGTAGTCCCAATCTACGGTA
GGGTGATTTGGCTCTCAAGAGTAGGACGTGGTGGGATCCAGTCACCAAGTCA
CGATTCTGTTCGTATGTGATAGTATCGCACAACTCAAGGACTTTCCTCTGACC
TGCTGTATCACTTACCATGCCAATCGTGCGAACCAACAGCGGGTTTTGTCCAT
AAACGGGTTGTTGGCCTGGGCTCCTATCTCGTTGCCGCTGGAGACTAATGCTC
GCTTACATGTAGCATTCGGACTCCATTGGGTCCAGAAGTGTCCGTCCTTGTGA
GGAGGAACCGATACTAAGCATGCATCTTGGACCAGTGGACAGCATAGGATGC
ACATACACATCTCCGTAATGGTGGATTCAATAAGAGCTGTCATGTGATAGCC
CGTAACCTTAGCTGAGGTCCTGAGACGTGTGGTACGTCAATGACTTGATGTCG
AACCTGTTCTCCGAGTAAGTTTGGTCGTGTTCGACCAAAGGCTTCAGCCGACC
TTACAGCCTCTGTTTCGTCTCAGGCAACTCTTCCGAGGTCGGATTTTACCGAT
ACCTACGCTAGTATGTTTGCCACTCCAGAGATAACGTGTGGGAGACCACTAG
GGTCAGCAACCTTGGAAGCCACTGAGCAAGCAGGATCTTTGTCCCATCGAGG
TTAGGACACCTTTGTAACCTGTGAAACGGACACTTCATATGGCTCGTTTGCTA
GCCTTTGGTATCTTCTCGTGGTCGCAGTCGGAGTCCATAGCATTGTCGAAGGA
TCGTCCTTTGAAGTCTGGTCTCGAGAGGGACGGCTAGTATCCCATCATCCGGT
TTCATTAATCCGCGACCTTGGACAAGATTCACCTCTCGCTTCCAACCTAGATT
GTTTTCTAAGCCCGCGTTGGACTGTGTGAGTACAATGTCCGGTCTGCGTTTGC
TCCAGCTAGGGGATTCACGAGCTGTAGCTCAGCTAGAGTGCGTAAGAAGCAT
GCCTAACGTAGCCGTTGACATGCAGATCCCAAAGGTCCTGCTCGTCTCCTAGA
CTGGATTAAACGCGATATTCGCGTAGTCAGCTGCGTCCCCTGTCATCCTTACC
CTACGATACGACGCTCACACACAGTTACCCGCAGGAATCGTGCAGCCGTCCG
TAGAGAGCTTGGCGTTAATTCCTTAGATTTGTGTATCCCAAAAGCAACGCACG
AGCACCAGGACCTCGTGAGCTTTGACCGGTTTATGTGTTTTGATAGGAACACG
GGTCCCTAGACCTTGCACGATCCAAATGCCACACGAAAGGTGGCCATGAGTT

TCGTGACAGCGCCATATGGCACAATCATCTGATTCGGGCTAAGCATCTATCTT
CGTCGTGTCTGGTTGGCAGAACGTCGGATAATGGCTAATCGTAGGTTGAGAC
CTCCCAGGTTGGCACTGTAGCCGATTGCCAACCATTAGATTCGCTATCATGTG
CTCATGTAGGGGTCTTTGCTTGCCTGAGAGAAAGTGTCAAACGCCCTATTTGG
TATCTCATCGAGGTAGATATATAGACGCGTTATAATTGGGGGCCAAGTTCGTT
TTCGTAGGTGAAGATTTATCCTAGCATGTCAGGTCTAACCCAGGTTTATTGAG
GACACCTTCTCGTGTGAAGGCTCCTAGCTGTACACGCCTCCGTGTTACATTAT
GTGGCTTACCAGACTTGCCTACAATTCAGTAGTGTTCTTTCAAACTCCAGATC
CGTTGAGTTGACATGCCTACTGGTTCTGAGTACCCTTAGCTTTGGAGGATCCT
AGCAATGTAGGAATATCACTAATCCACACAGGCTTTACGCAAGCCTGACAGT
TCGTGCCATGTGGAAGATCTTCGAAGATCAGGTATCCTTTACGACTGGCTAAC
CGAACACTCAAGAGTGTAGGTGAATTGAGAGGCTCCGAAGTGAGTGATCGGG
ATAAGTACGAAGATCGTGGAGGCGAGCATGTTACCATATAGCTAAGTCGGCA
TGTGGTACGATGCTACACTCCGATTGCTCGGACACTTTTCTCCGCTAATACGT
CATCCTGCTAGTCCGTGATTTGTTTCGACAACAGACCCTAAGTTTTTTGGCAT
CGTCTGTACTCTCGGTCTTTGAGCCTCTTGCAACGAATCATAGACACTCAATA
GATCGGAACCTTTGCTTGTCGTTCCTCAGGCATACTTTTTTGACATGCTGAGG
TCTGGTTTCTTCTTCCTCCCTTTCGAGACGTACATGCAGAGAAGTTTGGTTACT
TTCTGGGTTTCACATGCTTCGGCGGATGCTCATACCGAAGACACTGTCACCTG
TTTCCTTTGTCGGTACCTCGACTCGTTTTTTGTCCACGAGGTAGGACGTTTGTC
CTAAAGGTGTGGACTCCATCCAGGCGATTACAGCTAGGATTCGATGGTTGTTT
CGCCATCTCCTTGGTCCTTTTTTCGGGAAAGGAGCCAGGAAAACTTTTCGAAC
ATCAGAACCTGCTTGACGTCGCTATGCACGGATCGAACTTTGATTTACTCGTG
AAGACTTACAGATCCTAGGGCTTCGCAGTCAAGCTCATACCTTTGGACTAGTT
CCATGGACTTTTTTGAGTCTGGAACGTACGTGCACGTTTAGCATGCAGTGTAT
CCTAGCCCAAATGCAGAAGATCCTGCCAATTTGTGCGATTCAGACTGTGACA
GGTTTTTTGGCTTAACAGTCAGCAGTTTCATCCGTGAACTTTAGCTCCCAGGG
AGGTAGGTAGTCGTTGTAGCCTAGGTTGTTTACGCACCTATCCACCACAAGTG
TGTCCAGCTCCGTATTCTAGCTTTAGTGCTAGCTTGAGTGGAGACCTTTTTTCT
GTGACACTCACTGGGTTTGAAGAAAATAGAGACAACGAGGCTCCATGCATTA
GGGTGACCCTCTTGTGTCGTTTGTACGCGTGGACCTGGAT

9 crossing number square knotted RNA
GGGAGAGGAUCCAGAUGAUGUCUCUAUGGCCAAAAGUUGAAGGUCCGACU
ACACGUUGCGUAACGGUAAGCACUCAUAUGAGUAUGACAGGUCAUAGCAG
UAAAACGUCUAUAGUCCAGCAGGAAACUGCUGGAUAGGUUGGGCAGUGCA
ACUAGGUCGACGAAAGUCGAGUUGCCUAGUAUGAAAAGAACAGUCGUCUC
GUGAUGUCCAGCGAGUAGCAGUAUCUUAGAGAGUCGAUUCUCGCACUGGG
UUGAAAAUCCAAGUAACACUUUGCGAAAGCAAAGUGGGUAACUCAAGGUU
UCAUCUCCACCACGAAAGUGGUAUGUGUCUGCCUUAAAAUUGCGGUGCUU
CAUAAGUGAUCGAUCGUUACUGUCAGCUCCAGACCUUUACCACUGUGUCA
GACAGAAAAUGGAUACGGGUCGUUACUCACCGCUUUAGUAGUGAUCAGUA
CUUCUACUUAAGACCACUAGAUGCUAAAAUCUGGCCCAAAAGAUUUGGAU
UUGACCCAUGUCCUUACUGAUUACUCAUUCUCGUCAUCCGAGUUCAAAACU
ACGUAGGAAUGCAUCAUGCAGAACUACUGAGCGAUCCAAUAAUGGUUCAG
GAGUGUGUAUAUCUAAAAGCGUGUCUAGCAGGUGUGGCUGGGUACACGUC

UCGAAUAGACGAGCGACGUUGGGCACUAUACGGUAAAACUGUGACACUGU
UGUCGGAAACGACAACAGACCUCAAGAGAUUAGCAACGUGCACGGAAACG
UGCGUCGCAAAGCUACAAAAUCUAGUGCACCUUAGGAAUGGUCAUAAUAG
AUGCCGUCCAGCUGUCAUGACACCUAGGGUCACUCCAAAACUCUCUAACUG
GUACGAGAAAUCGUACCACUGACCAAGGGAACUAGAGACAGUGUCCUUCU
AGAAAUAGAAGGACACACAUGAACGUUCCAAAACUUGGUCAGUCUCCAGC
GAAAGCUGGAGAGUUAGAGAGGGAGUGACCCUAGGUUAAGUAGAAGCUGG
ACGGCAUCUAUUAUGACCAAGUAACGAGUGCACUAGAGUAGCUUUGCGAC
AACGGGAAACCGUUACGUUGCUAAUCUAAAACUUGAGGUCCAUGUAUCGA
AAGAUACAUGGUGUCACAGACCGCUGACACACCAACGUCGCUCGUGAGCUG
ACGACGUGUACCCAGCACUUAUGACUAGACACGCAGAUAUACACACUCCAU
CGACUCUAUUGGAUCGCUCAGUAGUUCUGCAAUCACGAGUCCUACGUAGG
AACUCGGAUGACGACAUACUCAAAUCAGUAAGGACAUGGGUCAAAUCUGU
AGUCGUUGGGCCAGAAGCAUCUAGUGGUCUGUCAUGACAGUACUGAUCAC
UACUAAAGCGGUGUUCCUAAGCCCGUAUCCACUGUUAUAGUGCGUGGUAA
AGGUCUGCUAUUCGAAGUAACGAUCGAUCCACACCUGAGCACCGCAAAAG
GCAGACACAUGUGCAGAAAUGCACGGAGAUGAAACCUAAAAUGAGUUACC
CCAACGAAGAAAUUCGUUGGUUACUUGGACAACCCAGUGCGAGAUGAACC
AUUCUAAGAUACUGCUACUCGCUGGACUGAUGCAUACGACUGUUCCAUAC
UAGGCAACCGUUCGAAAGAACGCCUAGUUGCACUGAAAACCCAACCUAGCC
GUAGAGAAAUCUACGGCCUAUAGACGACUGCUAUGACCUGUGAAUGAGUU
AUGAGUGCUUACCGUUACGCAACGCAAAUCUUGACCUUCAACGGCCAGUUC
AUGUUCAUCUGGAUCUUCUCGAG
15 crossing number DNA tetrahedron
ATCGGACCTTACATCAGAACACACGAGGTCCTAATGTGGTGCTTCGTGTGGA
GCCAGATTAGGCCTAAAATCTTGAACACGGGTCTCAGTAATTGATGATGCTG
TCTGAGAGCATGCCACAACTACGGGAGGGTAGGCCCCTTGACATCCTTTCTG
GGTTCACCAAATAGGGGATGTGTTAGGATGTCTCGGTTCTTAAGCTATTGGTC
TTCTTGATTGTCAGCCTGTCGTTCATAACAGACGAGTAACTGTGTTGGCACGG
CACGTCTGTTGTCGGAATCCTGCACAACATTCTGTTGACTTTCCCAAGACGAC
CTTCCAGTGCTGCTAAATGTATCTAGCCTCAACTTGCAAGGCGCGTACCGCGA
ATCGAATGCGTTTTCCATCTGGAAAATTGTGTGAAACGTTATGACTATTGCGC
ACGAAATGTAGAGCTCAGACCTCACGTGCGTTCGTTTGACAATAGGTGGTCG
TAGTTGCCATGACGTGGCACTGTCCGAATCGGACGTGCAGCCTGCATTAGCT
GGCTTATTTAGATTTCTGAAGCCGGCTGTTGGCAGTTCTCCCGATTACAATAG
CCCAACAGTGCACGTGGGCACGGCCTCTGGTAGGGCATGTGCGATTGTCGAA
AGTGGGACCAAGCTCTCTCGTCGGTCCGCCTGGAGGTTTGGACCCATAAGTTT
GCGAGAAATCACTGAGCTCTCTTTCTCAACACCCAGACATCCAACGTTACGTA
CGACAAGTCTTGCAGCCATAGTTTCGTGGATCTCAGGGAGCCATTCAAATCC
GACTACTGGGATAGAGTCTGGTTCTATTAGACGTAGACCGGTGATGCCTATA
AAGCAGGGGTAGTTGCGGCGACTGGAAGGCTCGAAGTGCCAATTGATTTTCA
GGCTGATCTAGTTTTTCTAGATCAGCCTGAAAATCAACGTTACCTTCGAGCCT
TCCAGTCGCCGGGTGTACCCCTGCTTTATAGGCATCATTTCCGGTCTACGTCT
AATACCGTCAGACTCTATCCCAGTAAGACGATTTGAATGGCTCCCTAGACTCC
ACGCTATGGCTGCAAGACTTGTCGTACGTGGTCGGGGATGTCTGGGTGTTGA
GAAAGAGAACCTCATGATTTCTCGCCTTATGGGTCCAAACGAAGACGCGGAC

CGACGAGAGCGAGACGTCCCACTTTCTCCCATCGCACATGCCCTACCAGAGG
TTTCCGTGCCCACGTGCACTCACCGGCTATTGTAATCGGGAGAACGTAACGC
AGCCGGCTTCAGAAATCTAAATAAGTTTCCAGCTAATGCGCAGGACACGTCC
GATTCGGATGCCGTCACGTCATGGCAACTCGTCTGACCTATTGTCCGAACGCA
CGTGAGGTTGAGGTTCTACATTTCGTGCGCAATAGTCATCCGACCTCACACAA
TTTTCCAGATGGACGCATTCGATTCGCGGTACGCGCCTTCACACGTGAGGCTA
GATACATTTAGCATGTGTGGAAGGTCGTCTTGGGGTCAACAGAATGTTGTAG
GCTGTTCCGACAACAGACGCAGTGCGCCAACACAGTTACTACGACCTTATGA
ACGACAGGCTTTTGACAATCAACTCCAGCAATAGCTTAAGAACAGCTTGATC
CTAACACAGACACTATTTGGTGAACCCAGGGATGTCAAGGGGCCTACCCTCG
AACAGTTGTGGCATGCTCTCGTCGAGCATCATCAATTACTGGAGACCGTGTTC
AAGTTTATTTTAGGCCTAATCTGGCTCGCAAGTAAGCACCACATTAGGACCTC
GGCACTTCTGATGTAAGGTCCGAT

20 crossing number DNA pyramid
ATCTTGACTGGAATAACTTGTCGATCCTCGTGTGGCGTTTGTCAGGGTAGTAC
AGGTGCGGACTCAGGAACGATGTATCGCCATACACCAAGCTACAGTCTCCAA
AGGAAAAGCGTTGCTTAGTCACCCCGACGTTCGGACCATGATGCGGGTGAAA
ATGCAACGTTCTCCGTAGTGATCTCGGTGAGCTTGGTACGCCAGTGAAGTGC
GACGGACTAGTCGGATCGTTTGTATCCACTTACCAGCAATAACAAAGTGACG
ACATCAAAACGTGCGACCGCACAGACCAGTGTTCTGTCCGCTAATTGACGTA
CCACTACCTAATCGAAACATACCTGCTCGTATGTGCTCGTGGATGCTTGCGGA
ACTGTTATGGGCTTTCTCTTTAGCGGCGATGTCTCGGCTATGGAGTTTTGGAG
TTCTGCACCGGCCGGTGTGGTGTGGCAAAGATCCCATTTACTTCTCTGCCCAG
TGGCTGTTATCAAGCCGAGCGTCAACCCAAACTTAACTGGCAGTGCTAACTG
CACTGGGTTCTCTCACTTAAATGGACCTCGGCTCGGGCAGCTGGGCCACTCGA
ATCTGCGCTCCCAGATAAACTCGACCCATCACTTGCATAGGTGGGCTGTCGTG
ACTAGCATGTTATCCTCAAGCTCGGACTCGGGATCCCGTGTTCCGCACAATTC
GGGCTTGAGGGCACGTGTCCCATAGGTACCTACGCTGGAGAAAACAGATCTG
CGAAGGACAGGCTATTAGCTTTAGATCTCTGTGGCACAACGGGTTGCCATTG
GAGCTGGTATAAGCATAGCTCGCATCTAGGCCCAAGTCTCTTCTAGGTTCTTC
CTCGCGTCGAGAGAAGTATAAATCGCACCCAATCCATAATACCCAACCCGGC
ATAAAGTCCTCAAAGGATTACTGCAACTGTTACTGCTGATTCTCGGAAATGTG
ACGGTAGTTACGTACGGTACCAGACCCTTGACAATTTCGATTGGGTCCGGGTT
CTTATCTTGATCACACTTTTCATGATACCTATGTGTACACAGCCTGAGCCTTA
ACTAGTTTGACGGGAAAACACAATAGACGCACTGTTTTCAGCGAAATAGGAC
CTGAGAGGACTTTGTCAAGCATGGCTGCTTTGAGCCGTTGGGAATCAGTGTTT
ATGGACGGATCTTGCACCTGACGTCTCTCACCTAAAGGTTATCTAGTTTTTCT
AGATAACCTTTAGGTACTCGACGTCAGGTGCAAGATCCGTTCGTTGACACTG
ATTCCCAACGGCTCAAAGCATTTGCCATGGTCCACAAAGTCCTCTCAGGTTAC
CTTTCGCTGAAAACAGTGTACGTATTGTGTTTTCCCGTCCTAGTTAAGGCTCA
GGTGGGAGACACATAGGTATCATGAAAAGTGTGCGAGCCATAAGAACCCGG
ACCCAATCGTTGTCAAGGGTCTGGTACCGTATGCGACTACCGTCACATTTCCC
GTCATCAGCAGTAAGCTGGTCAGTAATCCTTTGAGTTTGACTTTATGCCGGGT
TGGGTACAACGAATTGGGTGCGATTTATACTTCGAGTGACGCGAGGAAGAAC
CTAGAAGTTTAGACTTGGGCCTAGATGTCCACTATGCTTATAGAGCAGCCAAT

GGCAACCCGTGTAGTGACAGAGATCTGCTAATAGCCTGTCCTACTGCCATCTG
TTTTCTCCAGGACGCTTACCTATGGGACACGCACTGGCAAGCCCGAATTGTGT
TTCGGAACACGGGATCCCGCAGGCGAGCTTGAGGATAACATGCTCTGTGTGA
CAGCCCACCTATGCAAGTGATGGGTTTTCGAGTTTATCCTGTGTCGCAGATTC
GAGTGGCCCAGCTGCCATCAAGGAGGTCCATTTAAGTGAGAGAACCCATTTG
TGCAGTTAGCTCGCAGAGTTAAGTTTGGGTTCGTAGGCGGCTTGATAACAGCT
GCCCTGCAGAGAAGTTGGGATCTTTGCCACACCACACACCTCGGTGCAGAAC
TCCAAAACTCCATAGCGATCACATCGCCGCTAAAGAGGCCCATAACAGTTCC
GCAAGCACGAGCGAGCACATACCCAGCTGTATGTTTCGATTAGTGTGCCGTA
CGTCAATTAGCGTTTGACAGAACACTGGTCTGCGTAGTCGCACGTTTTGATGT
GAGAACTTTGTTATTCAGTTGAAGTGGATACCGATCCGACTACTTGGTCGCAC
TTCACTGGCGCCTAAAGCTCACCGAGATCACCGTCGAGAACGTTGCATTTTCA
CCCGTTTCATCATGGTCCGAACGTCGGGACACAGAAGCAACGCTTTTCCTTTG
GACCTGGTAGCTTGGTGTATGGCGATACTTTATCGTTCCTGAGTCCGCCGGCG
TACTACCCTGACAAACGCCACACGAGCGAGGACAAGTTATTCCAGTCAAGAT

22 crossing number DNA triangular prism
ATCCACAGTAGATGCGCTTTCCGCTGTCGAAGCGACTAGAATCGATAACCTC
GTCGCCTAAATTCTAGCGATCTTTCTCGTGGGTATGTAAGCAAGGACCGAGTC
CATAGTTGAAATCGGACACTTCTAGTCCTCTGGGCATAAACAGAGTCGACTG
GTCTCGTACATACTGTCAAACGAATGCCTGTAGAGGATACCTCCACCTACGAT
ACCATAAGGATAGACATTTTCTGTTACACCTATTCATCACCGGTTTGGCTACG
TCCGTGCAGCCGTTCGTGAGTAAGACCACATCTGACGCGGGAGAAAGCGCAT
GTGCCAAGTACAGACCTAGCAAGCCGGACGGATGTAATTCTGCCTAGGAGCA
CCAGGCTACGATCGATTCGCGTTTATAGCCTCTTATCTCGAGCTCGTCTCGTC
TGCCACGTACTTCCGCGCGGGTATATAGACACTTTGCCAACGTGCTGAGCGTT
TCCATAACTAGGATATATGTGGGTTCCAGTAGAGAGGGACAAGTCCTGGTTG
ATGGTTTTATCGCCACTGGGTCCAGCGAACCACCGATCAGCAGGATCACATC
TGCAGTAGACGCGAAAGAGGTCGGGACTCCCGTATAAGTGCTAGGCACCAAA
CGCATTTAAGAATCCGGACGGTCTGCCGGATACTCGTTCACCGTAGCCAAAA
TGGCACCTGAGGCTCAAAACCACGACCGCGAACATAAGAATACTTACGCTGC
CATGGGACGGGTATCCCTGACACTTAGGAGGCAGTCGATAAAGAAGTGCAGT
GACTGCCTATCACCCTTGGTCTGCCCGTCTGTTAGACTCTATATTCTGACTACC
CAGTGGTCACGATACCGGTGCCAGCCTACGGGTCTGAAGCCAGGAGTCGATG
CTGAATTGTCCTTTGCGATACAGATATGAACGACGTGCTAGGCGCTGTGGAG
TCCTACTGTACTACGGGATGTATACGTGTGCCTAGACAATAAAGGTAAGCTA
TGCTACCATCGCAGGACTATTCACTAACGTCTTTCCTGAGCTACATACCGATC
GATAGAGTTGCCTTTTAGTTTAGATGAACGTCACCTAAATATCTTCCAGACAG
GCCGAGAACTGGCACGCATCTTCGTATTTAGTCGACGGATGGGCTCCTTTGAT
CCTGTTGGATTACGACTATGGTACCAAAGAACGATCTCTATGAGATCCACTTT
CTCCGTGAAGAAACAATCCCGCACGAGGCAACTACGTTTACCCTGTTGGAGA
GCACTTGCTGCCTGCGCGTCAAACCGTCAGAGGGTGGCATCTGAGACGTTGT
ACCGGATTGATGGGAGAGTAGCGCCCAGCCGAGTTCTAGTTTTTCTAGAACT
CGGCTGGGCCACGCTCTCCCATCAATCCGGTACAGTTTGGCAGATGCCACCCT
CTGACGGTTTGACTTTGCGCAGGCAGCAAGTGCAGGTCAACAGGGTAAACGT
AGGCATCTCGTGCGGGATTGTTTCGAGACGGAGGTGGATCTCATAGAGACCA

TGGTTTGGTACCATAGTCGTAATCCAACGTCGTGAAAGGAGCCCATCCGTCG
ACTTACGAAGATGCTGTGCAGTTCTCGGCCTGTCTCTGCGATATTTAGGTGAC
GTTGGTGTAAACTAAAAGGCAACTCTATCTTTGATCGGTATGTAGCTCAGGA
ACCAAACTAGTGAATAGTCCTGCGATGGCGTGATAGCTTACCTTTATTGTCTA
GTTTGCACACGTATCGCGGACGTAGTACAGTAGGAAGACGAAGCGCCTAGCA
GAGGGTTCATATCTGTATCGCGGACAATTCAGCATCTCGCGTTGGCTTCAGAC
CCGTTCCTGCGCACCGGTATCGTGATGGACCGGTAGTCAGAATATAGTTTAGT
CTAACAGACGGGCATGATAAGGGTGATAGGCAGTCACTGTCCCAGTTTATCG
ACTGCCTCCTAAGTGTCAGTTTGGATACCCGTCTCGTTCCAGCGTAAGTATTC
TTATGTTCGCGAGGATCGTTTTGAGCCTCAGGTGCCATTTTGGTTTCTACGGT
GAACGAGTATCCGGACACGGGTCCGGATTCTTAAATGCGTTTGGTAGGAGCC
ACTTATACGGGAGTCTTTCCGACCTCTTGACTCCCTACTGCAGATGTGAAGGC
TGTGATCGGTGGTTCGCCCACTGCAGTGGCGATACCATCAACCAGGACTTGTC
CCTGCGAACTGGAACCCACATATATCCTAGTTATCAGCACGCTCAGCACGTTG
GCGTGTCTATATACCCGACATCCAGTACGTGGCAGACGCTCCACGCTCGAGA
TAACGTCCTATAAACGCGAATCGATCGTATTTGCCTGGTGCTCGCTCCTAGAA
TTACATCCGTCCGGCTTGCTACCGTGTTACTTGGCACATGCGCTTTCTCCCGCT
TTGTCAGAGTGCGTCTTACTCACGAACGGGGAAACGGACGTAGCCAAACCCA
TCATGAATAGGTGTAACAGATGTCTATCCTTATGGTATCGTTCTCGGAGGTAT
CCTCTACAGTTGCTCGTTTGACAGTATGTACTTCACCAGTCGACTTTTCTGTTT
ATGCCCAGAGGACTACTGGGATCCGATTTCAACTATGGACTCATCACTTGCTT
ACATACCCACGAGAATTTAGATCGCTAGAATTTAGCTCTCGAGGTTATCGATT
CTAGTCGCTTCGAGGAAGGAAAGCGCATCTACTGTGGAT

25 crossing number DNA pentagonal pyramid
ATCATGCGTGAGCCGGACTCCTGTACTCATTGCTAATGTACCTATGGCTAAGG
AGTCGGGTACACATATCTCTTTCCCGATAAACACATCTGCGATTCGGAAGCCC
GTCAGCTTTCGATCGCTCTGATCACACCGGAGTTGGCTCTTGCTTCGTATAGT
GCAGAAAGTGCGAATTGAGTAACCTCTCTCACGAATGAGAAGACACTACTGC
GTGTCGTCAGTTGGGATTTCCGCTGGGTACACAGCTTCGGGTACGCTCTATTC
ACTGCCCATACGCGGCTAGTGCCTGGAAAGCAATTCAAACGTCACGGAGTCT
CCTCACGTGGGATGAGCACCCACGTGACTAACGAACCACTGGGACGCGGCTC
GACTTTGAAGTTTCATCTTTAAGCCCAGTAAACCGCAGCTTCAAACGAAATGT
TACACGACACATCCATCGCTCATGAACAAACGCATTTGTACTCCACTAGACC
GGATCCTTACTTTTCCGACGGTTTCCAATTGCCTGCCAAGACACAATCTAACG
TCCTCGGAACCCTTAGCGACGCACATAGGTCCCTCGTGGGACCAACCCGAAA
GGAGTATGGAGAGTGTCTTTTGGTGGGATTTAGGACCTCGCGTCCACACATCT
GCATTTGTACCGCGGGTGTAAGTCACAGGGCTTTTCGGGATCACTTGCTAACT
TTTCCCTATTGCTATTACGAACAGGCAGACATATGAAAGGCCACCAGTCGGC
ATCCGGTTGCTGCACGTCACTTGCTTTCCGGTTGTTCTCCTGGTAACAACATG
TTGGCTCGTGAGTATGCAACTGTCCAACTGTCCAGTAAAGATCTTCTGATGCC
AAATCGTTCCTGGACTTCATTCAGTTGTATCAGTACTTACCTGGTACGTTTGTT
GAAAACAAGCCTTATGCACACTTTACCCAGTTACGAACGGTCATGACTCTCC
GTTAAGAGAGAAGAGTGATTTGCATCGGAAATGGACGTTTAAGGACCTTAGA
GTAGTAAGCCATAAGACAGAGAACGAGAGGAATGGAGCTGAGCCGACATAT
TCCACTGACAAGCAATGCCAGCCGTGTGCTGCGGCAATAGTTAACTCAGCTC

ATGCTACACTGGCCTCTTGATTAAACCTCTGACAAAAGCCGCACGGACTGGG
CACAGTAGCCGTAGCGTGTGATGTTCGACTGTGCACCAGAGCTTTTGGTAAC
GCTTTTAGGTAGACGGGAACCGGGAAAACTGTGTGACATGTTAACCAATCTG
CCATATACGAGGAACGTCCCGAAGTGACTTTGCAGAACATCATACAGCTCCA
TGACTGGCACGTCCGCGAAGTCGGTTCGACGCACCTTGGTTGGTTTCCGTCCC
TTATAATGTGGTGGAGTACCAGTAGCAATAAGTCGGGTTCCTAGGCTCGCAG
AGTTCTATCCATGTGCCGACTATGGGACCGTCTCAGCAGGGAGTAGGTACGA
GACCCTGACCCTCGGGCAGTGGGAATCTGCGTTCTCTAGTTTTTCTAGAGAAC
GCAGATTCATGTTGCCCGAGGGTCAGGGTCTCGTACCTAGGTTCTGCTGAGAC
GGTCCCATAGTCTTTGGCACATGGATAGAACTAGTGGAGCCTAGGAACCCGA
CTTATGATCCATGGTACTCCACCACATTATAAGGGACTTTGGAAACCAACCA
AGGTGGTGTGAACCGACTTCGCGGACAGGTCAGTCATGGAGCTGTATCTGCT
TCTGCGTCACTTCGGGACGTTCCTCGTATATCACTGGTTGGTTAACATGTCAC
ACAGTTTTCGTTGCACCCGTCTACCTAGCGTTACCAAAAGCCAGGCAGCACA
GTCGAATGGGACACGCTACGGCTACTGCCGACAGTCCGTGCGGCTTTTGTCA
GTTTAGGTTTAATCAAGAGGCCAGTTGGATCTGAGCTGAGTTAACTATTGCCC
ACTCACACGGCTGGCATTGCTTGTCTTTAGTGGAATATGTCGGCTAGTGTCCA
TTCCTCTACGTGGCTGTCTTATGGCTTAGAGGAGTAAGGTCCTTCGTCCATTT
CCGATGCAAATCAGGCATCTCTCTTAACACCGTCTCATGACCGTTCGTACGGT
CTTAAAGTGTGCATAAGTTTGCTTGTTTTCAACAAACCGCTCAGGTAAGTACT
GATACAACTGAATGACTTGCAGGAACGATTTGGCATCAGAATTTGATCTTTAC
TCTCTCCTTGGACAGTTGCATATGGTCCAGCCAACATGTTGTTTGCGTCAGAA
CAACCGGGCAAGTGACGTGCAGCTGCAACATGCCGACTGGTGGCCTTTCATA
TGCCAGTGTGTTCGTAATAGCAATAGGGAGTTAGCAAGTGATCCCGAAAAGC
CCTTCAGAGTACACCCGCGGTACAAATGCACCGATGTGGACGCGAGGTCCTT
CCCACCAAAAGACAGGACAGATACTCCTTTCGGGTCTCACGCACGAGGGACC
TATGACCAGGGCTAAGGGTTCCGAGGTTTACGTTAGATTGTGTCTTCTCTGGC
AATTGGAAGGAGAGGGAAAAGTAAGGATCACTGGGAGTGGAGTACTGCGTT
TGTTCATGAGCGATGGCCACGTCGTGTAACATTTCGTTTGAAGCTGCCTCCTA
CTGGGCTTAAAGATGCTTCAAAGTCGAGCCGCGTCCCCAGCGTTCGTTAGTCC
GTTCTGTGCTCATCCCACGTCTACTCACTCCGTGACGTTTGTTTAATTGCTTTC
TCTGGTCTAGCCGCGTACATCCAGTGAATAGAGCGTACTGCCAGCTGTGTACC
CAGCGGTCCCAACTGACGACACGCAGTACGTCCTTCTCATTCGTGAGAGGTG
CTACTCAATTCGCACTTTGATGACTATACGAAGTTTCAAGAGCCAACTCCGGT
GTGAGTGACTCGATCGAAAGCTGACGGGCTTGATGATCGCAGATGTGTTTAT
CGGGATTTAAGAGATATGTGTACCCCAAGCCTTAGCCATAGGTACATTAGCA
ATGAAGCGAGGAGTCCGGCTCACGCATGAT

Topological control strands:
1:
GAACAGGTGAGCTCATAATGGCGTACGTTCGTACCCATTTTCGTAGACACTCC
TCAGTTTTTTCAAGAGAGTGGGCGTGTTGAGACTACAACAGGTTTTTTGTCAC
TGTAGTAGATCTTTTCCTGGCTTAAATCAGGTCGCCGGCATCTGATACTGGCA
TCAGGCTGTGACGGACAAAATCAACTTTTGACAAAGAGCACAGGGTTTTTTG
ATACTGCTCGAGCTCTCGGGAAGCGAGTTGGTTTTTTGAAGTTCGCTTTCGGG
TTTTGCAAGATAAGAGGCACCCTAGCCTCAGCGCAGCAATTATTCGTTGTTGA

CGAAACGCAGTCCGTTTTCTCCAAGGTACATAGGTTTTTTGCAACGTACCCAT
GGTCCTTGACATGTTTAGGTTTTTTGCGTGACATGTGTCGGTTTTAAGAGTGG
TGGACACGGACGTCACCTTGAAGTCTGATGCACAACTCTGGACCCATGTGTA
TCATTTTAGATACGAGCAATCCGTTTTTTGAGGGTGCTCGCCCGTGGAAGAGA
CAGTGCGGTTTTTTCTCTCCTGTCTTGGCATTTTGGACTTCTCGTGCTTCCACA
ATGACC

2:
CACCTGTTCGGTCATTGTGACACGTCGAGAAGTCCTGCCACTGTACGAGAGTT
TTTTCCGCAGTACAGCTTCCTTTTACGGGCCTTCTCCCTCTTTTTTCGGATAGA
AGGTATCTTGATACACATGGGTCGTTCGATGTGCATCAGACTTCAAGGTGAC
GTCGAATTGCACCACTCTTCCGACTACACGCACGCTTTTTTCCTAACGTGTAC
AAGGTTTTACCATGCCAGGGTTGCTTTTTTCCTATCCTGGTTGGAGCGGACTG
CGTTTCGTGCTGGCCGAATAATTGCTGCGCTGAGGCTAGGCGTTGGCTTATCT
TGCCCCGATGGATAACTTCTTTTTTCCAACTATCCACCCGATTTTGAGCTCTCT
GCGTATCTTTTTTCCCTGGCAGATTTGTCGTTGATTTTGTCCGTAGTCCGCTGA
TGCCAGTATCAGATGCCGGCGAGTCACGTTAAGCCAGGGATCTCGATTGGTG
ACTTTTTTCCTGTCAATCGCTCAATTTTCACGCCACCAGTCTTGTTTTTTCTGA
GCTGGTTCTACGTGGGTACGAACGTACAGACAGATGAGCT

3:
CATGACGGAGATTACCTCGAACTCCAGCTCGGATAGGTTTTGAGGACTACAA
CGTGTAATATTTTGCCTAAGGGTTAGCAATCCTGTCTAGCTAAAACACGTAGT
TTTCGAGCTCTGACGTGACGCCATCGATACCTCAGCGTATCGCCTCGGACTCT
ACCACAGAGGGTATTTTCCTTATGCGCCCAACGGTGTGTGGCTCCGTGCACCA
ATTCCTGCCAGCGTTGCTTACAGCGACTTTTGCGCTCGACTCAATTCTCCACT
GATC

4:
TCCGTCATGGATCAGTGGACGTGTCAGTCGAGCGCGTCGCTGTAAGCAACCA
ACAAAGGAATTGGTGCACGGAGCCACACACGTGCCTGCGCATAAGGTACCCT
CTGTGGTAGCACAGCAGGCGATACGCTGAGGTATCGATGGCCCTGATTCAGA
GCTCGCTACGTGTTTTAGCTGCCATTGATTGCTAACCCTTAGGCAAAATATTG
AAGCATGTAGTCCTCCCTATCCGAGCTGGACAGAGTGGTAATC

Reference:

1. G. Aad *et al.*, Search for a new resonance decaying to a W or Z boson and a Higgs boson in the [Formula: see text] final states with the ATLAS detector. *Eur Phys J C Part Fields* **75**, 263 (2015).
